# Cytokines and Abnormal Glucose and Lipid Metabolism

**DOI:** 10.3389/fendo.2019.00703

**Published:** 2019-10-30

**Authors:** Jie Shi, Jiangao Fan, Qing Su, Zhen Yang

**Affiliations:** ^1^Department of Endocrinology, Xinhua Hospital, Shanghai Jiaotong University School of Medicine, Shanghai, China; ^2^Shanghai Key Laboratory of Children's Digestion and Nutrition, Department of Gastroenterology, Xinhua Hospital, Shanghai Jiaotong University School of Medicine, Shanghai, China

**Keywords:** cytokine, glucose metabolism, lipid metabolism, insulin resistance, inflammation

## Abstract

Clear evidence indicates that cytokines, for instance, adipokines, hepatokines, inflammatory cytokines, myokines, and osteokines, contribute substantially to the development of abnormal glucose and lipid metabolism. Some cytokines play a positive role in metabolism action, while others have a negative metabolic role linking to the induction of metabolic dysfunction. The mechanisms involved are not fully understood, but are associated with lipid accumulation in organs and tissues, especially in the adipose and liver tissue, changes in energy metabolism, and inflammatory signals derived from various cell types, including immune cells. In this review, we describe the roles of certain cytokines in the regulation of metabolism and inter-organ signaling in regard to the pathophysiological aspects. Given the disease-related changes in circulating levels of relevant cytokines, these factors may serve as biomarkers for the early detection of metabolic disorders. Moreover, based on preclinical studies, certain cytokines that can induce improvements in glucose and lipid metabolism and immune response may emerge as novel targets of broader and more efficacious treatments and prevention of metabolic disease.

## Introduction

Over the decades, overnutrition coupled with a sedentary lifestyle has led to a striking increase in metabolic diseases, such as type 2 diabetes (T2D) and non-alcoholic fatty liver disease (NAFLD). Some organs and tissues (e.g., adipose, liver, muscle, skeleton) secrete specific cytokines for inter-organ communication, and the production and secretion of these cytokines alter during nutritional stress and physical activity. Recent studies have shown that certain factors participate in glucose and lipid metabolism (1–4), and thus may associate with metabolic disorders.

**Figure 1:**
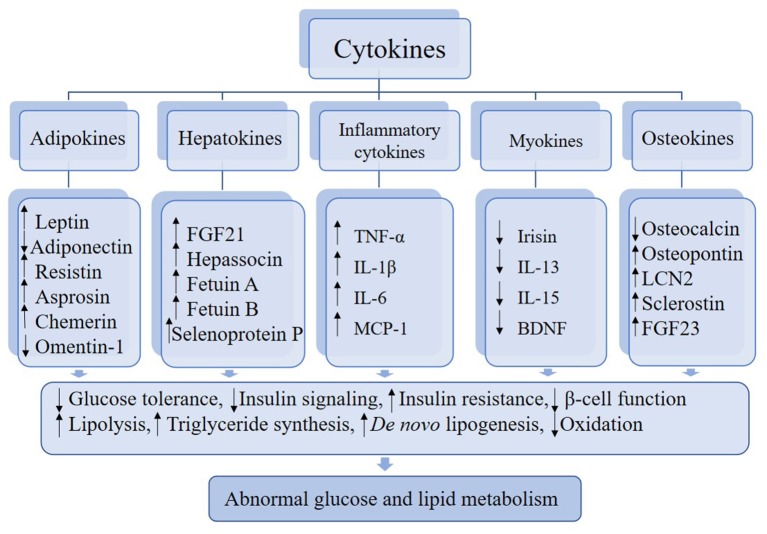
Alterations of cytokines levels and metabolic dysregulation.

In this review, we describe certain cytokines that are involved in abnormal glucose and lipid metabolism (Figure 1). Based on the most recent literature, we delve into the roles of these cytokines in the regulation of metabolism and inter-organ signaling (Table 1) with particular focus on the relation to pathophysiological aspects of metabolic disease. Finally, considering the emerging data supporting the contributions of various cytokines to metabolic disorders, we discuss the potential for these factors to emerge as biomarkers for the early detection of metabolic disorders and as novel approaches for therapy.

**Table 1:** Cytokines that involved in glucose and lipid metabolism.

**Cytokines**	**Metabolic actions**	**Circulating levels**	**References**
**Adipokines**			
^a^Leptin	Promotes the oxidation of fatty acids, enhances insulin sensitivity, stimulates the uptake of glucose, controls feeding	↑obesity,↑T2D	(5–7)
^a^Adiponectin	Enhances insulin sensitivity, anti-steatotic, anti-inflammatory, anti-fibrotic, regulates healthy longevity	↓T2D,↓NAFLD	(8–12)
^b^Resistin	Induces insulin resistance, increases lipid secretion	↑T2D	(9, 13–16)
^b^Asprosin	Accelerates hepatic glucose production, increases food consumption and body weight	↑obesity,↑T2D	(17–20)
^b^Chemerin	Exacerbates glucose intolerance, impairs insulin signaling and vascular dysfunction	↑T2D,↑NAFLD	(21–28)
^a^Omentin-1	Promotes glucose uptake, improves insulin sensitivity, anti-atherosclerotic	↓T2D, ↓MetS	(29–33)
**Hepatokines**			
^a^FGF21	Ameliorates pancreatic β-cell function and survival, increases glucose uptake, maintains glucose homeostasis, inhibits lipolysis	↑obesity,↑T2D	(34–40)
^b^Hepassocin	Blocks insulin signaling, induces insulin resistance, exacerbates lipid accumulation	↑NAFLD, ↑T2D	(41–43)
^b^Fetuin A	Causes insulin resistance, pro-inflammatory	↑T2D	(44–53)
^b^Fetuin B	Induces glucose intolerance and insulin resistance	↑NAFLD,↑T2D	(54, 55)
^b^Selenoprotein P	Impairs insulin signaling and glucose homeostasis, increases glucose output	↑NAFLD,↑T2D	(56–59)
**Inflammatory cytokines**			
^b^TNF-α	Mediates insulin resistance, stimulates of lipolysis, pro-inflammatory	↑T2D	(60–67)
^b^IL-1β	Stimulates triglycerides, cholesterol accumulation, and lipid droplet formation; reduces insulin-stimulated glucose uptake and lipogenesis	↑obesity,↑T2D, ↑NAFLD	(68–80)
IL-6	Has a dual role in modulating insulin action	↑T2D	(62, 81–89)
^b^MCP-1	Induces insulin resistance, elevates hepatic triglyceride content	↑T2D	(90–95)
**Myokines**			
^a^Irisin	Induces glucose and fatty acid uptake, ameliorates hepatic steatosis, improves insulin resistance, anti-inflammatory, loses weight	↓obesity,↓T2D	(96–103)
^a^IL-13	Increases skeletal muscle glucose uptake, oxidation, and glycogen synthesis	↓T2D	(104, 105)
^a^IL-15	Enhances insulin sensitivity and action, reduces fat mass and adipogenesis, decreases circulating triglycerides and VLDL	↓obesity	(106–110)
^a^BDNF	Enhances insulin signal transduction and fat oxidation	↓T2D	(111–116)
**Osteokines**			
^a^Osteocalcin	Promotes β-cell proliferation and insulin expression and secretion, favors glucose uptake and utilization in muscle, favors fatty acid uptake and utilization in muscle	↓T2D, ↓MetS	(4, 117–124)
^b^Osteopontin	Induces steatosis, inflammation, insulin resistance, and gluconeogenesis	↑NAFLD	(125–130)
^a^LCN2	Improves insulin sensitivity, decreases body weight and fat mass	↑obesity,↑T2D	(131–135)
^b^Sclerostin	Enhances *de novo* lipid synthesis and reduces fatty acid oxidation	↑T2D	(136–139)
^b^FGF23	Mediates insulin resistance, stimulates lipolysis	↑T2D	(140–147)

a *Cytokines that induce positive metabolic effects*.

b *Cytokines that induce negative metabolic effects*.

*↑Increased circulating levels*.

*↓Decreased circulating levels*.

## Adipokines

The adipose tissue is not only an inert repository of excess energy but also a complex, highly active metabolic endocrine organ that secretes numerous cytokines, which are collectively termed adipokines (148). The endocrine functions of adipose tissue are induced by secreted proteins stimulated by metabolic effects and enzymes involved in the metabolism of steroid hormones (148). Most, but not all, adipokines are peptides or proteins with hormone-like properties that signal the functional status of adipose tissue to targets in the brain, liver, muscle, pancreas, immune system, and other tissues (149). A portion of the adipokines have been confirmed to directly or indirectly affect glucose and lipid metabolism, as well as insulin sensitivity through modulation of insulin signaling (148).

## Leptin

Leptin, the product of the obese gene (*ob*; also known as *Lep*), is an adipocyte-derived hormone, which is responsible for the regulation of feeding behavior and energy homeostasis through the central nervous system (CNS) (150, 151).

Leptin promotes the oxidation of fatty acids through its stimulation of Adenosine 5′-monophosphate (AMP)-activated protein kinase (AMPK) phosphorylation and activation (5). It also enhances insulin sensitivity in the peripheral tissues, which is mediated by the central activation of the phosphoinositide 3-kinase (PI3K)/Akt pathway (6). Moreover, leptin stimulates the uptake of glucose and prevents the accumulation of lipids in non-adipose tissues, which can result in functional impairments known as “lipotoxicity” (7). The leptin deficient mice (*ob/ob* mice) exhibited hyperphagia, obesity and insulin resistance, while the administration of leptin in leptin lacking mice reverses these alterations (152).

In humans, the congenital leptin deficiency leads to significant hyperphagia, early-onset extreme obesity, and hormonal and metabolic disturbances (153). Consistent with mice studies, administration of recombinant leptin effectively improved metabolic disorders in patients with lipodystrophy or congenital leptin deficiency (154, 155). Notably, leptin concentrations are significantly increased in obesity and T2D (156), and positively correlated with adipose mass, indicating the occurrence of leptin resistance (157). Further investigations and experimentations need to be done to shed light on molecular mechanisms of leptin resistance.

Leptin exerts potent anti-diabetic actions, independent of its effects on body weight. Indeed, long-term leptin administration could significantly improve glycemic control, insulin sensitivity, and lipid metabolism in mice with T2D (8, 158). However, data from clinical trials failed to find that leptin can effectively improve insulin sensitivity in T2D people with severe obesity (9, 159). Nevertheless, due to the fact that not all T2D subjects are overly obese, an issue is: does administration of leptin improve insulin sensitivity in non-obese, leptin-sensitive, T2D individuals?

## Adiponectin

Adiponectin is a peptide predominantly expressed in white adipose tissue (WAT), and also produced in hepatocytes during stress (10, 11). Contrary to other adipokines, adiponectin is negatively associated with fat mass (160).

The powerful insulin-sensitizing role of adipokines is due, in part, to its binding to cognate receptors, such as adiponectin receptor (AdipoR)1 and AdipoR2, subsequently leading to activation of AMPK and peroxisome proliferators-activated receptors (PPAR)-α signaling pathways (10). Moreover, adiponectin has an anti-steatotic effect on the hepatocytes, due to increases in free fatty acid (FFA) oxidation, and reduces FFA influx, *de novo* lipogenesis and gluconeogenesis (12). Notably, adiponectin protects hepatocytes from apoptosis, a hallmark of NAFLD, by inhibition of c-Jun NH2 terminal kinase (161). In addition, adiponectin exerts anti-inflammatory and anti-fibrotic action though acting on HSC, Kupffer, and possibly sinusoidal cells (162). In mice, administration of adiponectin exhibits glucose-lowering effects and improves insulin resistance, while adiponectin-deficient mice suffer from insulin resistance and diabetes (163). More recently, a study reported that AdipoR1 regulates healthy longevity through the activation of AMPK in skeletal muscle, which in turn activates SirT1 (13). Similarly, another study in *C. elegans* showed that the adiponectin receptor (PAQR-2) signaling acts as a key player linking low temperature with autophagy to extend lifespan (164).

High adiponectin levels were associated with a markedly reduced relative risk of T2D (14). Circulating adiponectin levels, as well as those of AdipoR1/R2 expression, are decreased in the conditions of obesity, T2D and NAFLD (15). Given that the US Food and Drugs Administration has not yet approved any therapies for the treatment of NAFLD and disease management is concentrated on treatment of common comorbidities, adiponectin may be a promising therapeutic target for NAFLD. Further experimental investigations are needed to estimate the efficacy and safety of adiponectin therapy in patients with NAFLD.

## Resistin

Resistin (named after “resistance to insulin”) is a member of the family of resistin-like molecules (RELms), also known as “found in inflammatory zone” (FIZZ) (162). In mice, resistin is synthesized mainly in adipocytes (16), whereas in humans, resistin is predominantly produced by macrophages infiltrating adipose tissue and peripheral blood mononuclear cells, and it is not detectable in adipocytes (165).

Resistin has been shown to induce insulin resistance in mice (9). Cell-based studies revealed that resistin greatly increased hepatocyte very low-density lipoprotein (VLDL) apoB and lipid secretion through enhancing microsomal triglyceride transfer protein (MTP) activity, impairing intracellular insulin signaling and stimulating *de novo* lipogenesis via the sterol regulatory element-binding protein (SREBP)1 and SREBP2 pathways (166). Administration of recombinant resistin impairs glucose tolerance and insulin sensitivity in normal mice, whereas treatment of anti-resistin antibody improves these metabolic abnormalities (16). Mice lacking resistin have low post-fasting blood glucose levels via reduced hepatic glucose production (167). And resistin deficiency in ob/ob mice leads to increased obesity due the reduction in metabolic rate without an affect on food intake, but also leads to improved glucose tolerance and insulin sensitivity largely owing to enhancing insulin-mediated glucose disposal, as well as drastically attenuated hepatic steatosis (17, 18).

Resistin expression was increased in subjects with central obesity, T2D and NAFLD (19, 20). Although studies in animal models consistently show that resistin promotes insulin resistance, evidence for this effect in humans is unclear. Thus, further researches are required toward this direction.

## Asprosin

Asprosin, the C-terminal cleavage product of profibrillin (encoded by *FBN*1), is a new fasting-induced glucogenic protein hormone produced by WAT and associated with hepatic glucose release (168). Asprosin accelerates hepatic glucose production by activating the G protein-cAMP-protein kinase A (PKA) pathway (169). It also acts as an orexigenic hormone, that activates AgRP neurons to increase food consumption and body weight (170). In mice, a single injection of asprosin caused a swift rise in blood glucose and insulin levels, while a reduction in asprosin and treatment with an asprosin-specific monoclonal antibody improved insulin sensitivity and reduced appetite and body weight (168, 169).

In humans, asprosin-deficient patients showed a reduction in subcutaneous WAT and a unique pattern of metabolic disorders, including partial lipodystrophy, along with decreased plasma insulin while maintaining euglycemia (169). Asprosin concentrations are increased in conditions of obesity and T2D, and are independently associated with levels of fasting glucose and triglycerides (171). It remains unclear whether the asprosin inhibition could be effective management for obesity and T2D. It has not yet been determined that the receptor for asprosin is involved, and the factors regulating its secretion are not clear. Further research is needed to fill these gaps.

## Chemerin

Chemerin is an adipokine secreted in an inactive form (prochemerin) and activated through C-terminal cleavage by inflammatory and coagulation serine proteases (172), acting through its receptor, such as the chemerin receptor (ChemR) 23 (21). Although chemerin and its receptors exist throughout the human body, the adipose tissue and hepatocytes are major sources of chemerin (22, 23).

Plasma chemerin levels were found to be increased in diet-induced obese mice, and in another study of obese diabetic db/db mice, the chemerin was found to exacerbate glucose intolerance, lower serum insulin levels, and decrease tissue glucose uptake (24, 25). Importantly, ChemR23 knockout mice presented with reduced adiposity and body mass, and the chemerin levels were shown to be reduced by weight loss and fat reduction (26). In addition, antagonism of the chemerin/ChemR23 system in a T2D animal model was found to ameliorate vascular dysfunction and normalize insulin signaling via redox-sensitive and Akt-dependent pathways (27). However, a study reported that isolated islets and perfused pancreas from chemerin-deficient mice revealed impaired glucose-dependent insulin secretion, and conversely, chemerin transgenic mice revealed enhanced insulin secretion and improved glucose tolerance (28).

In humans, the circulating chemerin concentration is significantly elevated among individuals with obesity and/or T2D (173) and NAFLD (29), and the levels of chemerin correlate with levels of pro-inflammatory cytokines, such as tumor necrosis factor (TNF)-α and interleukin (IL)-6 (30, 31). Targeting chemerin/ChemR23 may be a potential therapeutic strategy to improve insulin resistance and vascular function in obesity-associated diabetes.

## Omentin

Omentin-1, also known as intelectin-1, is a novel adipokines mainly expressed in visceral adipose tissue and is the major circulating form of omentin (32). The levels of omentin-1 is downregulated by glucose and insulin, and upregulated by fibroblast growth factor-21 and dexamethasone (33).

Omentin-1 is known to have key roles in the maintenance of body metabolism and insulin sensitivity, and has anti-inflammatory, anti-atherosclerotic, and cardiovascular-protective effects via AMP-activated protein kinase/Akt/NF-κB/mitogen-activated protein kinase (ERK, JNK, and p. 38) signaling (33). In cultured human vascular cells, exogenous omentin promoted glucose uptake, and improved the insulin activity and anti-inflammatory response (174).

In humans, serum omentin-1 levels are significantly decreased in patients with obesity (54), T2D (175), and metabolic syndrome (MetS) (34). And plasma omentin-1 levels were inversely correlated with body mass index (BMI), waist circumference, leptin levels, and insulin resistance, and positively correlated with adiponectin and high-density lipoprotein (HDL) levels (54). Thus, circulating omentin-1 levels may serve as a biomarker of related metabolic disorders.

## Hepatokines

It is well-established that the liver is a crucial organ in energy stores, including systemic glucose and lipid metabolism (35). Hepatic fat content is an excellent marker of the metabolic abnormalities, and hepatic steatosis has a causal role in the induction of a series of metabolic disease, such as NAFLD, MetS, and T2D (1).

Hepatokines are proteins either uniquely or predominantly produced by the hepatocytes; upon secretion, certain hepatokines influence metabolic processes via autocrine, paracrine, and endocrine signaling pathways in the liver and in non-hepatic tissues (1). Under some circumstances, especially the condition of liver steatosis, the hepatokine production and secretion profile can be altered (36). These factors show positive or negative metabolic effects, with some improving metabolic variables, while others lead to metabolic dysfunction and inflammation (37).

## Fibroblast Growth Factor (FGF)21

FGF21, a member of the FGF superfamily, is a secreted protein expressed mainly in the liver (38). In general, it serves as a metabolic regulator and is known to induce positive metabolic functions that regulate insulin sensitivity and lipid and energy metabolism (39).

In adipose tissue, FGF21 inhibits lipolysis (40) and increases insulin-dependent glucose uptake via up-regulated expression of glucose transporter 1(39). FGF21 preserves pancreatic β-cell function and survival by activation of the extracellular signal-regulated kinase (ERK) 1/2 and Akt signaling pathways (152). Moreover, FGF21 induces fasting gluconeogenesis via the brain-liver axis, to maintain glucose homeostasis during prolonged fasting (176). FGF21 knockout mice showed severe hypoglycemia and defective hepatic gluconeogenesis, and these impairments reversed after injection of FGF21 (176). In obese mice, administration of recombinant FGF21 has been shown to alleviate hepatic steatosis, induce browning of WAT, increase energy expenditure, improve insulin sensitivity, and restore glucose tolerance (39, 177).

Circulating FGF21 levels are elevated in subjects with obesity, T2D (37), and MetS (41), and positively correlate with triglycerides, fasting insulin, and insulin resistance (41). Serum levels of FGF21 is a sensitive marker of the degree of steatosis (42). Consistent with the mice studies, the administration of FGF21 analog shows good performance in patients with obesity and T2D (43, 44). Hence, the favorable metabolic action of FGF21 treatment appears to be in contrast with the elevated levels of FGF21 detected in obese and T2D subjects. It remains unclear whether these conflicting findings suggest that high levels of FGF21 are produced in these cases to compensate for underlying metabolic stress, or whether FGF21 resistance is present in the context of high levels of FGF21 associated with obesity and T2D.

## Hepassocin

Hepassocin, also called fibrinogen-like protein 1 and hepatocyte-derived fibrinogen-related protein 1 (HFREP1), is a liver-specific growth factor that has been found to participate in the regulation of proliferation of hepatocytes and regeneration of the liver (45). High glucose regulates the expression of hepassocin, and the fasting glucose concentrations is an independently associated factor for the plasma hepassocin levels (46).

Cell-based studies in HepG2 hepatocellular carcinoma cells revealed that hepassocin can block insulin signaling and induce insulin resistance through an ERK1/2-dependent signaling pathway (46). In mice, both the hepatic over-expression of hepassocin and administration of recombinant hepassocin lead to exacerbated hepatic lipid accumulation and induction of insulin resistance in both liver and skeletal muscle tissues (46). Conversely, knockdown of hepassocin in HFD-fed mice led to improved glucose utility and insulin sensitivity, and ameliorated impaired insulin signaling both in liver and skeletal muscle (46).

In humans, circulating levels of hepassocin are increased in prediabetes, T2D, and NAFLD, owing to its association with impaired fasting glucose, glucose intolerance, and insulin resistance (46, 47). Consequently, high hepassocin levels are risk factors for insulin resistance and diabetes, and hepassocin may be a promising biomarker for the detection of prediabetic status.

## Fetuin A

Fetuin A is a glycoprotein expressed predominantly in the liver and has been identified as an endogenous inhibitor of insulin receptors (48). This glycoprotein is an independent risk factor for the development of T2D (49).

Fetuin A impairs insulin action working through its binding to the insulin receptor tyrosine kinase in liver and skeletal muscle, and resulting in decreased rates of autophosphorylation and downstream insulin signaling cascades (48). Fetuin A also stimulates the production of pro-inflammatory cytokines in adipocytes and macrophages (50). The process involves Fetuin A serving as an endogenous ligand for the Toll-like receptor (TLR) 4, which then enables free fatty acids to activate TLR4 signaling to induce insulin resistance (51). Besides, liver expression of the gene encoding fetuin A positively associates with the expression of key enzymes in glucose and lipid metabolism (52). Mice that knocked out the gene encoding Fetuin A (*Ahsg* gene) were insulin sensitive (53) and resistant to weight gain when fed a HFD (178). Whereas, injection of recombinant fetuin A into mice reduced insulin sensitivity (179).

In humans, circulating levels of fetuin A are elevated in patients with hepatic steatosis (55) and T2D (52); moreover, this increase is correlated strongly and negatively with insulin sensitivity (180). In view of the interaction between increasing plasma concentrations of both FFA and fetuin A resulting in insulin resistance (56), disruption of the fetuin A-TLR4 association may serve as a potential therapeutic strategy for T2D.

## Fetuin B

Fetuin B is the second member of the fetuin family, an endogenous inhibitor of the insulin receptor tyrosine kinase, and is produced primarily in liver tissue (57). Cell-based studies have demonstrated that fetuin B can lead to insulin resistance in myotubes and hepatocytes (36), and *in vivo* studies have shown that administration of fetuin B to lean mice causes glucose intolerance but not insulin resistance (36). Moreover, partial silencing of fetuin B in obese mice improved glucose tolerance, independent of weight loss (36).

In humans, plasma fetuin B levels are increased in obese individuals with hepatic steatosis (36) and T2D, and associated positively with intrahepatic triglyceride and insulin resistance (36, 58). However, the mechanism underlying its role in these pathogeneses remains unclear. Further researches are necessary to shed light on how fetuin B exerts its metabolic effect.

## Selenoprotein P

Selenoprotein P, encoded by the *Sepp*1 gene, is a secretory protein produced and secreted primarily by the liver. It is responsible for transporting selenium from the liver to extrahepatic tissues (59). A recent study reported that selenoprotein P regulates insulin action and systemic energy metabolism in rodents and humans (181).

Cell-based studies revealed that administration of purified selenoprotein P resulted in impaired insulin signaling through reduced insulin-stimulated phosphorylation of the insulin receptor and AKT in mouse primary hepatocytes and immortalized myocytes *in vitro*, and increased phosphorylation of insulin receptor substrate 1 (IRS1) at Ser307 (182). In mice, the administration of selenoprotein P induced hepatic and peripheral insulin resistance, whereas both genetic deletion and RNA interference-mediated knockdown of selenoprotein P ameliorated insulin signaling and improved glucose tolerance (182). Additionally, high circulating levels of adiponectin were observed in *Sepp*1 knockout mice, revealing the occurrence of crosstalk between the hepatokine selenoprotein P and the adipokine adiponectin (183).

In humans, selenoprotein P is increased in patients with T2D (182) and NAFLD (181), and is positively correlated with triglycerides, glucose, and insulin resistance (184). However, most of current data comes from small sample clinical studies, thus further prospective large-scale studies are warranted.

## Inflammatory Cytokines

The immune system is closely linked to metabolic changes, and components are changed in obesity and T2D (68). The detection of immune cells in metabolic tissues and organs, such as macrophages, has highlighted a dynamic, ongoing crosstalk that exists between immune and metabolism regulatory systems (185). Their interactions are termed “immunometabolism.” Inflammation has emerged as an important pathophysiological factor of T2D, with inflammatory cytokines playing a pivotal role. Inflammatory cytokines characterize an inflammatory state (recently named as “metaflammation”), which is defined by a chronic low-grade inflammation initiated by metabolic and inflammatory cells in response to an excessive energetic nutrient load (186). Some pro-inflammatory cytokines, such TNF-α and IL-1β, take part in disrupting the insulin and lipid signaling pathways, thereby influencing insulin sensitivity and lipid metabolism (60). Promisingly, some treatments targeting pro-inflammatory cytokines displayed improved glucose metabolism and insulin secretion and sensitivity in T2D (61).

## TNF-α

TNF-α, a member of TNF family, is a potent pro-inflammatory cytokine and immuno-modulator produced mainly by macrophages and monocytes (62). TNF-α is involved with multiple functions and plays a variety of roles in metabolic disorders (63).

TNF-α is a mediator of insulin resistance through its ability to block the action of insulin (64). The TNF-α-mediated insulin resistance is partially through the down-regulation of key genes (i.e., Glut4), which are necessary for normal insulin action, direct influences on insulin signaling, induction of elevated free fatty acids by stimulation of lipolysis, and negative regulation of peroxisome PPAR-γ, a vital insulin-sensitizing nuclear receptor (65, 66). In mice, administration of exogenous TNF-α could lead to insulin resistance, conversely neutralization of TNF-α improved insulin sensitivity (67). Furthermore, TNF-α deficiency has been shown to significantly improve insulin sensitivity, and lower circulating levels of free fatty acids (187).

In humans, the circulating concentration of TNF-α are elevated in T2D, and this alteration is strongly associated with impaired glucose tolerance and enhanced insulin resistance, islet dysfunction, and increased T2D risk (109–111). Some studies have shown that a statistically significant reduction in the risk of developing T2D in treatment with TNF inhibition, and the improvement in insulin sensitivity in during prolonged treatment with the anti-TNF-α antibody infliximab in insulin resistant subjects (61). However, most of these studies were not prospective and it is unclear whether these improvements are direct or indirect influences. Further study is necessary to determine whether TNF-α inhibition can help in the management of human metabolic disease.

## IL-1β

IL-1β, a member of the IL-1 family, plays an important role in endocrinology and the regulation of responses correlated with inflammatory stress (72). IL-1β is considered to mediate inflammation, steatosis, and fibrosis in liver (68).

In β-cells, IL-1β activates the JNK pathway (73), which is involved in cytokine-mediated apoptosis (74) and mediates oxidative stress-induced suppression of insulin gene transcription (75). Moreover, IL-1β decreases expression of the insulin receptor substrate IRS-1, inhibits glucose transporter (GLUT)4 translocation to the plasma membrane, and reduces insulin-stimulated glucose uptake and lipogenesis (76). In mice, IL-1β was found to promote hepatic steatosis by stimulating triglycerides, cholesterol accumulation, and lipid droplet formation and to regulate inflammation, hepatic insulin resistance, and fibrosis (77). In contrast, inhibition of IL-1β was found to attenuate steatosis and liver injury (78), improve atherosclerosis (79), and lower glycemia (80). Furthermore, the IL-1β deficiency mice exhibited less hepatic steatosis and intact insulin sensitivity (188).

In humans, serum levels of IL-1β are increased in obesity, NAFLD (189), T2D (190), and MetS (81), but decrease significantly after extensive weight loss (82). Emerging clinical studies showed that IL-1 receptor antagonist (anakinra) and IL-1β-specific antibody (gevokizumab, canakinumab, LY2189102) treatment improved glucose metabolism and insulin secretion in patients with T2D (61, 83). Moreover, IL-1 antagonism was well-tolerated with no evidence of drug-related adverse events, apart from reactions at the anakinra injection site (61).

## IL-6

IL-6 is a multifunctional cytokine with complex roles and is expressed in numerous cells, including immune cells, skeletal muscle cells, and islet β-cells (84). It has a dual role in modulating insulin sensitivity, acting as both an enhancer and inhibitor of insulin action (62).

To exert its biological effects, IL-6 utilizes two pathways: classic and trans-signaling. While it is generally believed that the classic signaling is participated in the anti-inflammatory and regenerative activities of IL-6, the trans-signaling is involved in the pro-inflammatory responses induced by this cytokine (85, 86). Moreover, IL-6 is responsible for macrophage recruitment to adipose tissue in obesity, leading to the development of inflammation, insulin resistance, and T2D (87). In addition, IL-6 has been shown to exert long-term inhibitory effects on the gene transcription of IRS-1, GLUT4, and PPAR, along with a marked reduction in IRS-1, and on insulin-stimulated tyrosine phosphorylation and insulin-stimulated glucose transport, which result in impaired insulin signaling and action (88). It is worth noting that the central application of IL-6 suppresses feeding and improves glucose tolerance via enhanced trans-signaling in the CNS of obese mice, even under conditions of leptin resistance (89). Studies have also shown that short- and long-term administration of adipocytes with IL-6 produces different influences on insulin signaling. Short-term treatment with IL-6 has been found not to impair the effect of insulin in the adipose tissue of rats (191), but increased glucose uptake in adipocytes (90). In contrast, chronic administration of IL-6 has been found to induce insulin resistance, suppress glucose transport, and reduce insulin-induced lipogenesis (88, 91). In humans, circulating levels of IL-6 are elevated in T2D, and this cytokine is an independent predictor of T2D (92).

## Monocyte Chemotactic Protein 1 (MCP-1)

MCP-1, a member of the chemokine (chemotactic cytokine) family, is a powerful monocyte agonist that plays a crucial role in the recruitment of macrophages (93). MCP-1 is mediated by NF-kB activation and oxidative stress (94), and up-regulated by oxidized lipids, endoplasmic reticulum stress (95), and high glucose concentrations (192). Additionally, MCP-1 links obesity to insulin resistance and hepatic steatosis (96).

Mice that were engineered to express the MCP-1 transgene showed macrophage infiltration into adipose tissue, elevated hepatic triglyceride content, and insulin resistance (96). MCP-1 induces hepatic steatosis and insulin resistance via up-regulating the expression of *SREBP-*1*c*, a transcription factor that regulates the expression of genes important in lipid synthesis, and glucose-6-phosphatase (G6Pase), an enzyme involved in hepatic glucose production (96). In contrast, MCP-1 knockout mice and inhibition of MCP-1 activity exhibited improvements in insulin resistance and hepatic steatosis (96, 97). Consistent with the findings in mice, humans show increased plasma levels of MCP-1 in T2D (98).

## Myokines

Skeletal muscle is considered to be the largest organ in the body of non-obese subjects and is now recognized as an active endocrine organ due to its function in releasing numerous myokines (3). Myokines are part of a complex communication network within the body which connects skeletal muscle with other organs, such as adipose tissue, liver, and pancreas (3). Recent studies have indicated that myokines, such as irisin, interleukin (IL)-13, and IL-15, are deeply involved in glucose and lipid metabolism via autocrine, paracrine and endocrine activities (99). It is speculated that the contractile activity influences skeletal muscle secretory functions, which may link physical activity to the health-promoting effects of exercise [5].

## Irisin

Irisin, encoded by the *Fndc*5 gene, is the cleaved and secreted product of the fibronectin type III domain-containing protein 5 (FNDC5). This myokine is a transmembrane protein expressed principally in skeletal muscle (100), but is also expressed in WAT to a lower extent (101).

Irisin participates in energy expenditure via stimulation of the browning of WAT (100), direct induction of glucose and fatty acid uptake, and regulation of gene expression of metabolic enzymes in human muscle via AMPK activation (102). Cell-based studies revealed that the treatment of recombinant irisin greatly increased uptake of glucose and fatty acids, as well as expression of genes involved in glucose transport and lipid metabolism; however, the expression of genes involved in glycogenolysis or gluconeogenesis was suppressed (102). Furthermore, exogenously administered irisin, adenovirus-over-expressed irisin and irisin transgenic mice exhibited improved glucose homeostasis, ameliorated hepatic steatosis, improved insulin resistance, reduced inflammatory cytokine production, and weight loss (100, 103, 104, 193). *FNDC*5 gene deficiency aggravated fat accumulation, obesity, insulin resistance, and inflammation accompanied with enhanced AMPK inhibition, macrophages recruitment, and M1 polarization (103).

In humans, irisin levels, and *FNDC*5 gene expression are decreased in obese and T2D individuals (105). A previous study reported that irisin increases immediately after exercise and is positively correlated with exercise intensity (102). The positive role of irisin in metabolism supports the idea that moderate exercise is good for health. However, there are some conflicting findings in the literature (194). To date, the role of irisin in T2D is still not entirely clear. Further research is required to determine the links between irisin and T2D.

## IL-13

IL-13 is well-known as an anti-inflammatory cytokine by inhibiting the secretion of some inflammatory cytokines derived from macrophages and monocytes (106). Recently, however, IL-13 was determined to also serve as a novel myokine that is synthesized and released by human myotubes under the conditions of accelerated glucose uptake and metabolism through autocrine pathway (173).

It has been demonstrated that IL-13 is a master regulator of glucose metabolism, working via suppression of hepatic glucose production and directly inhibiting the transcription of hepatic genes that encode key gluconeogenic enzymes, such as *PEPCK* and *G*6*P* (107). In cultured human myotubes, the levels of IL-13 were found to be significantly decreased (~75%) in those from T2D individuals compared to myotubes from heathy subjects (108). IL-13 exposure increases skeletal muscle glucose uptake, oxidation, and glycogen synthesis via an Akt-dependent mechanism (108). On the contrary, genetic deletion of IL-13 in mice resulted in hyperglycemia, which progressed to hepatic insulin resistance and systemic metabolic disturbances (107). And basal glycogen synthesis was found to be reduced in cultured myotubes upon exposure to an IL-13-neutralizing antibody (108).

However, the molecular mechanisms underlying the regulation of IL-13 expression and release by exercise are, as yet, unclear. Further research should be undertaken to explore how exercise affects IL-13 expression and secretion.

## IL-15

IL-15, a member of IL-2 superfamily, is a myokine that is highly expressed in skeletal muscle and released by myotubes; it is also produced by a wide variety of other cells and tissues (109).

The IL-15 secreted from skeletal muscle communicates with the adipose tissue to stimulate a reduction in fat mass and adipogenesis, and to decrease triglycerides and VLDL in blood (110), primarily through the UCPs and PPAR-δ signaling pathways (195). Moreover, in skeletal muscle and liver, IL-15 may enhance insulin sensitivity (196) and subsequent glucose transport and utilization, thereby improving glucose homeostasis through the activation of GLUT4 via Jak3/STAT3 (197). It has been demonstrated in animal models and human that IL-15: improves lipid and glucose metabolism, and insulin sensitivity; enhances mitochondrial activity; reduces WAT inflammation; and alleviates endoplasmic reticulum stress (197). Genetic research reported that IL-15 transgenic mice exhibited lean body condition, whereas IL-15 gene knockout mice showed significant increase in weight gain without changes in appetite (111).

In humans, plasma IL-15 is significantly decreased in obesity (111) and negatively associated with fat mass (112). Therefore, IL-15 may be a feasible therapeutic target for prevention and treatment in obesity and T2D.

## Brain-Derived Neurotrophic Factor (BDNF)

BDNF, a member of the neurotrophic factor family, is a protein produced in skeletal muscle cells that is increased by contraction (113). BDNF regulates neuronal differentiation and synaptic plasticity, and its reduced levels are involved in the pathogenesis of Alzheimer's disease and other disorders (114).

It is reported that BDNF increases phosphorylation of AMPK and acetyl CoA carboxylase, enhances fat oxidation (115), regulates glucose metabolism, and ameliorates insulin sensitivity (116). In obese diabetic mice, BDNF reduces food intake and lowers blood glucose levels (131). In another study, the administration of BDNF enhanced insulin-triggered tyrosine phosphorylation of the insulin receptor in the liver (116) and insulin-stimulated phosphatidylinositol-3 kinase (PI3K)/Akt activity, which demonstrated that BDNF can enhance insulin signal transduction (116). Moreover, in db/db mice, the hypoglycemic effect induced by the administration of BDNF was found to last for several weeks after treatment cessation and was independent of food reduction (131). Conversely, BDNF-deficient mice displayed hyperphagia, obesity, hyperleptinemia, and hyperinsulinemia (198).

In humans, plasma BDNF is decreased in individuals with both obesity and T2D, and is inversely correlated with serum levels of free fatty acids and insulin resistance (199). Moreover, lower BDNF levels are involved with obesity and diabetic complications (200).

## Osteokines

Recently, bone has emerged as an unexpected pleiotropic endocrine organ according to the finding of its secretion of molecules, which act in autocrine/paracrine manners to modulate skeletal homeostasis as well as some extra-skeletal systems (201). Bone is not only metabolically active, with glucose as the major energy source, but also actively takes part in systemic energy metabolism (4). First, osteoblasts can uptake glucose (a process primarily mediated by GLUT1) for utilization through aerobic glycolysis (202). Interestingly, the total uptake of glucose by bone exceeds that of traditional glucose-utilizing organs, including muscle, WAT, and the liver (203). Second, numerous key enzymes associated with the glycolytic pathway in carbohydrate metabolism are also present in osteoblasts (204) and osteoclasts (136). Moreover, osteoblasts express both the insulin receptor (205) and GLUT (117), which provides the basis for bone-mediated regulation of glucose metabolism. In addition, several osteokines (bone-derived cytokines), such as osteocalcin (118), lipocalin 2(201) and sclerostin (119) link bone and glucose metabolism, with involvement in modulation of glucose homeostasis, appetite, and browning of adipose tissue.

## Osteocalcin

Osteocalcin, also known as bone γ-carboxyglutamic acid protein, is one of the osteoblast-specific proteins that is an established biomarker of bone turnover, and it is reported to be associated with glucose and fat metabolism (118, 120). Circulating levels of undercarboxylated and bioactive osteocalcin double during aerobic exercise at the time levels of insulin decrease (121).

The endocrine functions of osteocalcin are fulfilled by its undercarboxylated form, termed undercarboxylated osteocalcin (ucOC) (4). Osteocalcin receptors are present in both central nervous system (122) and peripheral tissues, for instance, in the pancreas (123), adipocytes (124), and muscle (121), thereby facilitating its physiological functions. There is a growing body of experimental evidence suggesting that ucOC promotes pancreatic β-cell proliferation and insulin expression and secretion (118, 123), induces up-regelation of adiponectin in adipocytes to ameliorate insulin resistance (124), promotes release of glucagon-like peptide-1 to indirectly stimulate insulin secretion (206), and favors glucose and fatty acid uptake and utilization in muscle during exercise (121). Mice lacking osteocalcin manifested decreased β-cell proliferation, glucose intolerance, and insulin resistance (118), In contrast, the metabolic abnormalities in these mice were improved by infusion of exogenous ucOC (125).

In humans, serum osteocalcin levels are significantly lower in subjects with T2D (126) and MetS (120). However, the results regarding incident T2D are controversial. In several longitudinal studies, the serum osteocalcin level was found to not associate with the development of T2D (127).

## Osteopontin

Osteopontin, a member of small integrin-binding ligand N-linked glycoproteins (SIBLINGs) family, is a major non-collagenous bone matrix protein which participates in normal and pathological calcification (128). This glycoprotein is expressed in a variety of cells, including osteoblasts, osteoclasts, macrophages, as well as T-lymphocytes (128). Osteopontin acts as a mediator of obesity-related hepatic alterations including steatosis, inflammation, insulin resistance, and excess gluconeogenesis (129).

Cell-based experiments have shown that osteopontin impaired differentiation and insulin sensitivity of primary adipocytes as determined by inhibition PPAR-γ, adiponectin gene expression and insulin-stimulated glucose uptake (130). Mice deficient in osteopontin have improved glucose tolerance and lower fasting plasma glucose, insulin, triglycerides, and proinflammatory cytokines after high fat diet regime compared to wild-type mice (129) and antibody-mediated neutralization of osteopontin action reduces obesity-induced inflammation and insulin resistance (207).

In humans, serum osteopontin may reflect up-regulated gene expression during liver fibrosis in NAFLD and may serve as a test for advanced hepatic fibrosis in NAFLD (208). Moreover, osteopontin is involved in the development of diabetic vascular complications (132, 209).

## Lipocalin (LCN)2

LCN2, a small secreted transport protein, was initially recognized for its role in innate immunity (133) and was then identified as an adipokine capable of inducing insulin resistance (134). More recently, a new insight on LCN2 was gained with the discovery of Lcn2 expression in mice being at least 10-fold higher in bone than that in WAT (201).

Mechanistically, osteoblast-derived LCN2 has been shown to cross the blood-brain barrier and suppress appetite after binding to the melanocortin 4 receptor (or MC4R) in the hypothalamus by activating an MC4R-dependent anorexigenic (appetite-suppressing) pathway, thereby decreasing body weight and fat mass and improving insulin sensitivity (201). Mice lacking *Lcn*2 specifically in osteoblasts, rather than in adipocytes, showed increased food intake, fat mass, and body weight, along with decrease in glucose tolerance, insulin sensitivity, and serum insulin levels after glucose or arginine load (201). Meanwhile, islet number and size, β-cell mass and proliferation, and insulin secretion were also decreased in the LCN lacking mice (201). Conversely, chronic administration of exogenous LCN2 to lean and obese mice produced similar effects, with food intake, fat mass and body weight gain becoming reduced and glucose metabolism and energy expenditure becoming heightened (201).

In humans, postprandial serum levels of LCN2 become significantly increased in normal-weight individuals after high-fat meals, and this effect is accompanied by enhanced total energy expenditure; the effect is opposite (decreased LCN2) in obese subjects (135). Intriguingly, LCN2 expression and serum levels are higher in obesity (210), T2D (137), and NAFLD (138). In addition, LCN2 levels were positively correlated with adiposity, hypertriglyceridemia, hyperglycemia, and insulin resistance index but negatively correlated with HDL cholesterol (139). As such, there may be a compensatory mechanism at the early stage of this disease.

## Sclerostin

Sclerostin is a secreted protein predominantly expressed in osteocytes and is inhibited by osteoblast differentiation and bone formation (211). In general, sclerostin is considered a local inhibitor of bone acquisition that antagonizes deep bone metabolism via Wnt/β-catenin signaling (119).

Sclerostin exerts profound control over skeletal and whole-body metabolism by regulating the Wnt/β-catenin signaling pathway (119).Serum sclerostin levels were increased in mice models of disturbed metabolism, while sclerostin-deficient mice and those treated with a sclerostin-neutralizing antibody exhibited a reduction in the accumulation of WAT, along with corresponding enhancements in glucose and fatty acid metabolism, and increased insulin sensitivity (119). In contrast, recombinant sclerostin treatment was found to enhance *de novo* lipid synthesis and reduce both fatty acid oxidation and the expression of genes associated with fatty acid catabolism (119).

In humans, circulating levels of sclerostin are increased in T2D (140) and positively associated with BMI and fat mass (141). Moreover, the serum sclerostin levels exhibit a positive correlation with fasting glucose and result in insulin resistance; but negatively correlated with whole-body glucose disposal and insulin clearance rate (142).

## FGF23

FGF23, a unique member of the FGF family, is derived from bone that acts as a hormone and regulates renal phosphate and vitamin D metabolism (143). A growing body of epidemiological and experimental evidence suggests that FGF23 may regulate lipid and glucose metabolism as well as insulin action (144), but the underlying mechanisms are unclear. Furthermore, FGF23 involvement has been implicated in the onset and progression of atherosclerosis via its effects on endothelial cell function (145).

FGF23 knockout mice presented with reduced fat mass, developed hypoglycemia and increased peripheral insulin sensitivity, and showed improved subcutaneous glucose tolerance (146), suggesting a link between FGF23 and insulin resistance. However, another study in FGF23 lacking mice demonstrated no influence on aging, glucose homeostasis, or lipid metabolism with a non-functioning vitamin D receptor (147), suggesting that FGF23 may exert its effects depend on functioning vitamin D receptor.

In humans, serum FGF23 levels are elevated in individuals with obesity (212), MetS (144), prediabetes (213), and T2D (214). Moreover, FGF23 is associated positively with triglycerides, BMI, waist circumference, and fat mass, and negatively correlated with HDL and apolipoprotein A1 (144). However, another cross-sectional study of small sample did not show differences in circulating FGF23 levels between diabetic and non-diabetic patients, but reported that circulating FGF23 is associated with bone mineral density and preclinical vascular disease in T2D patients (215). Further experimental studies are needed to shed more light on the underlying mechanisms between FGF23 and glucose and lipid metabolism, and prospective studies of large scale are needed to determine the association between FGF23 and metabolic disease, such as T2D.

## Conclusion

Overnutrition and physical activity alter cytokines secretion, thereby influencing metabolic and immune regulatory pathways that caused or promoted metabolic disorders. These cytokines are part of a complex network that mediates communication between multiple organs and tissues (e.g., adipose, liver, muscle, skeleton). The emerging data support the contributions of certain cytokines to metabolic disorders. Given the disease-related changes in levels of relevant cytokines (for instance, leptin, adiponectin, reisitin, FGF21, Fetuin A, TNF-α, IL-6, MCP-1), these factors may serve as biomarkers for the early detection of metabolic disorders. Moreover, based on preclinical studies, certain cytokines (FGF21, leptin, adiponectin, irisin) that can induce improvements in glucose and lipid metabolism and may emerge as novel targets of broader and more efficacious treatments and prevention of metabolic disease.

## Author Contributions

ZY, QS, and JF contributed conception of the paper. JS wrote the manuscript. ZY and QS revised the manuscript.

### Conflict of Interest

The authors declare that the research was conducted in the absence of any commercial or financial relationships that could be construed as a potential conflict of interest.

## Abbreviations

T2D: type 2 diabetes
NAFLD: non-alcoholic fatty liver disease
MetS: metabolic syndrome
CNS: central nervous system
FFA: free fatty acids
BMI: body mass index
HDL: high-density lipoprotein
VLDL: very low-density lipoprotein
HFD: high-fat diet
WAT: white adipose tissue
FGF21: fibroblast growth factor 21
IRS1: insulin receptor substrate 1
TNF-α: tumor necrosis factor α
IL: Interleukin
MCP-1: monocyte chemotactic protein 1
NASH: non-alcoholic steatohepatitis
SREBPC: sterol regulatory element-binding protein C
ApoB: apolipoprotein B
BDNF: brain-derived neurotrophic factor
ucOC: undercarboxylated osteocalcin
LCN2: Lipocalin 2
PPAR: peroxisome proliferators-activated receptors
PKA: protein kinase A
ERK: extracellular signal-regulated kinase
MAPK: mitogen-activated protein kinase
GLUT: glucose transporter.

## References

[1] Meex RCR Watt MJ. Hepatokines: linking nonalcoholic fatty liver disease and insulin resistance. Nat Rev Endocrinol. (2017) 13:509–20. 10.1038/nrendo.2017.56 28621339

[2] Cao H. Adipocytokines in obesity and metabolic disease. J Endocrinol. (2014) 220:T47–59. 10.1530/JOE-13-0339 24403378 PMC3887367

[3] Eckardt K Gorgens SW Raschke S Eckel J. Myokines in insulin resistance and type 2 diabetes. Diabetologia. (2014) 57:1087–99. 10.1007/s00125-014-3224-x 24676645

[4] Liu DM Mosialou I Liu JM. Bone: another potential target to treat, prevent and predict diabetes. Diabetes Obes Metab. (2018) 20:1817–28. 10.1111/dom.13330 29687585

[5] Minokoshi Y Kim YB Peroni OD Fryer LG Muller C Carling D. Leptin stimulates fatty-acid oxidation by activating AMP-activated protein kinase. Nature. (2002) 415:339–43. 10.1038/415339a 11797013

[6] Morton GJ Gelling RW Niswender KD Morrison CD Rhodes CJ Schwartz MW. Leptin regulates insulin sensitivity via phosphatidylinositol-3-OH kinase signaling in mediobasal hypothalamic neurons. Cell Metab. (2005) 2:411–20. 10.1016/j.cmet.2005.10.009 16330326

[7] Unger RH Zhou YT Orci L. Regulation of fatty acid homeostasis in cells: novel role of leptin. Proc Natl Acad Sci USA. (1999) 96:2327–32. 10.1073/pnas.96.5.2327 10051641 PMC26783

[8] Cummings BP Bettaieb A Graham JL Stanhope KL Dill R Morton GJ. Subcutaneous administration of leptin normalizes fasting plasma glucose in obese type 2 diabetic UCD-T2DM rats. Proc Natl Acad Sci USA. (2011) 108:14670–5. 10.1073/pnas.1107163108 21873226 PMC3167517

[9] Moon HS Matarese G Brennan AM Chamberland JP Liu X Fiorenza CG. Efficacy of metreleptin in obese patients with type 2 diabetes: cellular and molecular pathways underlying leptin tolerance. Diabetes. (2011) 60:1647–56. 10.2337/db10-1791 21617185 PMC3114380

[10] Kadowaki T Yamauchi T Kubota N Hara K Ueki K Tobe K. Adiponectin and adiponectin receptors in insulin resistance, diabetes, and the metabolic syndrome. J Clin Invest. (2006) 116:1784–92. 10.1172/JCI29126 16823476 PMC1483172

[11] Yoda-Murakami M Taniguchi M Takahashi K Kawamata S Saito K Choi-Miura NH. Change in expression of GBP28/adiponectin in carbon tetrachloride-administrated mouse liver. Biochem Biophys Res Commun. (2001) 285:372–7. 10.1006/bbrc.2001.5134 11444852

[12] Heiker JT Kosel D Beck-Sickinger AG. Molecular mechanisms of signal transduction via adiponectin and adiponectin receptors. Biol Chem. (2010) 391:1005–18. 10.1515/bc.2010.104 20536390

[13] Yamauchi T Kadowaki T. Adiponectin receptor as a key player in healthy longevity and obesity-related diseases. Cell Metab. (2013) 17:185–96. 10.1016/j.cmet.2013.01.001 23352188

[14] Spranger J Kroke A Mohlig M Bergmann MM Ristow M Boeing H. Adiponectin and protection against type 2 diabetes mellitus. Lancet. (2003) 361:226–8. 10.1016/S0140-6736(03)12255-6 12547549

[15] Tsuchida A Yamauchi T Ito Y Hada Y Maki T Takekawa S. Insulin/Foxo1 pathway regulates expression levels of adiponectin receptors and adiponectin sensitivity. J Biol Chem. (2004) 279:30817–22. 10.1074/jbc.M402367200 15123605

[16] Steppan CM Bailey ST Bhat S Brown EJ Banerjee RR Wright CM. The hormone resistin links obesity to diabetes. Nature. (2001) 409:307–12. 10.1038/35053000 11201732

[17] Qi Y Nie Z Lee YS Singhal NS Scherer PE Lazar MA. Loss of resistin improves glucose homeostasis in leptin deficiency. Diabetes. (2006) 55:3083–90. 10.2337/db05-0615 17065346

[18] Singhal NS Patel RT Qi Y Lee YS Ahima RS. Loss of resistin ameliorates hyperlipidemia and hepatic steatosis in leptin-deficient mice. Am J Physiol Endocrinol Metab. (2008) 295:E331–8. 10.1152/ajpendo.00577.2007 18505833 PMC2519749

[19] Gierej P Gierej B Kalinowski P Wroblewski T Paluszkiewicz R Kobryn K. Expression of resistin in the liver of patients with non-alcoholic fatty liver disease. Pol J Pathol. (2017) 68:225–33. 10.5114/pjp.2017.67583 29363914

[20] McTernan CL McTernan PG Harte AL Levick PL Barnett AH Kumar S. Resistin, central obesity, and type 2 diabetes. Lancet. (2002) 359:46–7. 10.1016/S0140-6736(02)07281-1 11809189

[21] Ferland DJ Watts SW. Chemerin: a comprehensive review elucidating the need for cardiovascular research. Pharmacol Res. (2015) 99:351–61. 10.1016/j.phrs.2015.07.018 26211950 PMC4859430

[22] Bozaoglu K Bolton K McMillan J Zimmet P Jowett J Collier G. Chemerin is a novel adipokine associated with obesity and metabolic syndrome. Endocrinology. (2007) 148:4687–94. 10.1210/en.2007-0175 17640997

[23] Krautbauer S Wanninger J Eisinger K Hader Y Beck M Kopp A. Chemerin is highly expressed in hepatocytes and is induced in non-alcoholic steatohepatitis liver. Exp Mol Pathol. (2013) 95:199–205. 10.1016/j.yexmp.2013.07.009 23906870

[24] Ernst MC Issa M Goralski KB Sinal CJ. Chemerin exacerbates glucose intolerance in mouse models of obesity and diabetes. Endocrinology. (2010) 151:1998–2007. 10.1210/en.2009-1098 20228173

[25] Roh SG Song SH Choi KC Katoh K Wittamer V Parmentier M. Chemerin–a new adipokine that modulates adipogenesis via its own receptor. Biochem Biophys Res Commun. (2007) 362:1013–8. 10.1016/j.bbrc.2007.08.104 17767914

[26] Ernst MC Haidl ID Zuniga LA Dranse HJ Rourke JL Zabel BA. Disruption of the chemokine-like receptor-1 (CMKLR1) gene is associated with reduced adiposity and glucose intolerance. Endocrinology. (2012) 153:672–82. 10.1210/en.2011-1490 22186410 PMC3275396

[27] Neves KB Nguyen Dinh Cat A Alves-Lopes R Harvey KY Costa RMD Lobato NS. Chemerin receptor blockade improves vascular function in diabetic obese mice via redox-sensitive and Akt-dependent pathways. Am J Physiol Heart Circ Physiol. (2018) 315:H1851–60. 10.1152/ajpheart.00285.2018 30216119 PMC6336978

[28] Takahashi M Okimura Y Iguchi G Nishizawa H Yamamoto M Suda K. Chemerin regulates beta-cell function in mice. Sci Rep. (2011) 1:123. 10.1038/srep00123 22355640 PMC3216604

[29] Kukla M Zwirska-Korczala K Hartleb M Waluga M Chwist A Kajor M. Serum chemerin and vaspin in non-alcoholic fatty liver disease. Scand J Gastroenterol. (2010) 45:235–42. 10.3109/00365520903443852 20095887

[30] Weigert J Neumeier M Wanninger J Filarsky M Bauer S Wiest R. Systemic chemerin is related to inflammation rather than obesity in type 2 diabetes. Clin Endocrinol. (2010) 72:342–8. 10.1111/j.1365-2265.2009.03664.x 19558533

[31] Lehrke M Becker A Greif M Stark R Laubender RP von Ziegler F Chemerin is associated with markers of inflammation and components of the metabolic syndrome but does not predict coronary atherosclerosis. Eur J Endocrinol. (2009) 161:339–44. 10.1530/EJE-09-0380 19497986

[32] Yang RZ Lee MJ Hu H Pray J Wu HB Hansen BC. Identification of omentin as a novel depot-specific adipokine in human adipose tissue: possible role in modulating insulin action. Am J Physiol Endocrinol Metab. (2006) 290:E1253–61. 10.1152/ajpendo.00572.2004 16531507

[33] Watanabe T Watanabe-Kominato K Takahashi Y Kojima M Watanabe R. Adipose Tissue-Derived Omentin-1 function and regulation. Compr Physiol. (2017) 7:765–81. 10.1002/cphy.c160043 28640441

[34] Jialal I Devaraj S Kaur H Adams-Huet B Bremer AA. Increased chemerin and decreased omentin-1 in both adipose tissue and plasma in nascent metabolic syndrome. J Clin Endocrinol Metab. (2013) 98:E514–7. 10.1210/jc.2012-3673 23303213

[35] Tilg H Moschen AR Roden M. NAFLD and diabetes mellitus. Nat Rev Gastroenterol Hepatol. (2017) 14:32–42. 10.1038/nrgastro.2016.147 27729660

[36] Meex RC Hoy AJ Morris A Brown RD Lo JC Burke M. Fetuin B is a secreted hepatocyte factor linking steatosis to impaired glucose metabolism. Cell Metab. (2015) 22:1078–89. 10.1016/j.cmet.2015.09.023 26603189

[37] Stefan N Haring HU. The role of hepatokines in metabolism. Nat Rev Endocrinol. (2013) 9:144–52. 10.1038/nrendo.2012.258 23337953

[38] Fon Tacer K Bookout AL Ding X Kurosu H John GB Wang L. Research resource: Comprehensive expression atlas of the fibroblast growth factor system in adult mouse. Mol Endocrinol. (2010) 24:2050–64. 10.1210/me.2010-0142 20667984 PMC2954642

[39] Kharitonenkov A Shiyanova TL Koester A Ford AM Micanovic R Galbreath EJ. FGF-21 as a novel metabolic regulator. J Clin Invest. (2005) 115:1627–35. 10.1172/JCI23606 15902306 PMC1088017

[40] Arner P Pettersson A Mitchell PJ Dunbar JD Kharitonenkov A Ryden M. FGF21 attenuates lipolysis in human adipocytes - a possible link to improved insulin sensitivity. FEBS Lett. (2008) 582:1725–30. 10.1016/j.febslet.2008.04.038 18460341

[41] Zhang X Yeung DC Karpisek M Stejskal D Zhou ZG Liu F. Serum FGF21 levels are increased in obesity and are independently associated with the metabolic syndrome in humans. Diabetes. (2008) 57:1246–53. 10.2337/db07-1476 18252893

[42] Mutanen A Heikkila P Lohi J Raivio T Jalanko H Pakarinen MP. Serum FGF21 increases with hepatic fat accumulation in pediatric onset intestinal failure. J Hepatol. (2014) 60:183–90. 10.1016/j.jhep.2013.09.003 24021426

[43] Lin Z Tian H Lam KS Lin S Hoo RC Konishi M. Adiponectin mediates the metabolic effects of FGF21 on glucose homeostasis and insulin sensitivity in mice. Cell Metab. (2013) 17:779–89. 10.1016/j.cmet.2013.04.005 23663741

[44] Gaich G Chien JY Fu H Glass LC Deeg MA Holland WL. The effects of LY2405319, an FGF21 analog, in obese human subjects with type 2 diabetes. Cell Metab. (2013) 18:333–40. 10.1016/j.cmet.2013.08.005 24011069

[45] Gao M Zhan YQ Yu M Ge CH Li CY Zhang JH. Hepassocin activates the EGFR/ERK cascade and induces proliferation of L02 cells through the Src-dependent pathway. Cell Signal. (2014) 26:2161–6. 10.1016/j.cellsig.2014.04.013 24768768

[46] Wu HT Ou HY Hung HC Su YC Lu FH Wu JS. A novel hepatokine, HFREP1, plays a crucial role in the development of insulin resistance and type 2 diabetes. Diabetologia. (2016) 59:1732–42. 10.1007/s00125-016-3991-7 27221093

[47] Wu HT Lu FH Ou HY Su YC Hung HC Wu JS. The role of hepassocin in the development of non-alcoholic fatty liver disease. J Hepatol. (2013) 59:1065–72. 10.1016/j.jhep.2013.06.004 23792031

[48] Auberger P Falquerho L Contreres JO Pages G Le Cam G Rossi B. Characterization of a natural inhibitor of the insulin receptor tyrosine kinase: cDNA cloning, purification, and anti-mitogenic activity. Cell. (1989) 58:631–40. 10.1016/0092-8674(89)90098-6 2766355

[49] Stefan N Fritsche A Weikert C Boeing H Joost HG Haring HU. Plasma fetuin-A levels and the risk of type 2 diabetes. Diabetes. (2008) 57:2762–67. 10.2337/db08-0538 18633113 PMC2551687

[50] Mukhopadhyay S Bhattacharya S. Plasma fetuin-A triggers inflammatory changes in macrophages and adipocytes by acting as an adaptor protein between NEFA and TLR-4. Diabetologia. (2016) 59:859–60. 10.1007/s00125-016-3866-y 26781474

[51] Pal D Dasgupta S Kundu R Maitra S Das G Mukhopadhyay S. Fetuin-A acts as an endogenous ligand of TLR4 to promote lipid-induced insulin resistance. Nat Med. (2012) 18:1279–85. 10.1038/nm.2851 22842477

[52] Haukeland JW Dahl TB Yndestad A Gladhaug IP Loberg EM Haaland T. Fetuin A in nonalcoholic fatty liver disease: *in vivo* and *in vitro* studies. Eur J Endocrinol. (2012) 166:503–10. 10.1530/EJE-11-0864 22170794

[53] Mathews ST Singh GP Ranalletta M Cintron VJ Qiang X Goustin AS. Improved insulin sensitivity and resistance to weight gain in mice null for the Ahsg gene. Diabetes. (2002) 51:2450–8. 10.2337/diabetes.51.8.2450 12145157

[54] de Souza Batista CM Yang RZ Lee MJ Glynn NM Yu DZ Pray J. Omentin plasma levels and gene expression are decreased in obesity. Diabetes. (2007) 56:1655–61. 10.2337/db06-1506 17329619

[55] Reinehr T Roth CL. Fetuin-A and its relation to metabolic syndrome and fatty liver disease in obese children before and after weight loss. J Clin Endocrinol Metab. (2008) 93:4479–85. 10.1210/jc.2008-1505 18728159

[56] Stefan N Haring HU. Circulating fetuin-A and free fatty acids interact to predict insulin resistance in humans. Nat Med. (2013) 19:394–5. 10.1038/nm.3116 23558619

[57] Olivier E Soury E Ruminy P Husson A Parmentier F Daveau M. Fetuin-B, a second member of the fetuin family in mammals. Biochem J. (2000) 350:589–97. 10.1042/bj3500589 10947975 PMC1221288

[58] Walker GE Follenzi A Bruscaggin V Manfredi M Bellone S Marengo E. Fetuin B links vitamin D deficiency and pediatric obesity: direct negative regulation by vitamin D. J Steroid Biochem Mol Biol. (2018) 182:37–49. 10.1016/j.jsbmb.2018.04.009 29684480 PMC6092561

[59] Motsenbocker MA Tappel AL. A selenocysteine-containing selenium-transport protein in rat plasma. Biochim Biophys Acta. (1982) 719:147–53. 10.1016/0304-4165(82)90318-X 6216918

[60] Dinarello CA. Immunological and inflammatory functions of the interleukin-1 family. Annu Rev Immunol. (2009) 27:519–50. 10.1146/annurev.immunol.021908.132612 19302047

[61] Donath MY. Targeting inflammation in the treatment of type 2 diabetes: time to start. Nat Rev Drug Discov. (2014) 13:465–76. 10.1038/nrd4275 24854413

[62] Sethi JK Hotamisligil GS. The role of TNF alpha in adipocyte metabolism. Semin Cell Dev Biol. (1999) 10:19–29. 10.1006/scdb.1998.0273 10355025

[63] Xu H Uysal KT Becherer JD Arner P Hotamisligil GS. Altered tumor necrosis factor-alpha (TNF-alpha) processing in adipocytes and increased expression of transmembrane TNF-alpha in obesity. Diabetes. (2002) 51:1876–83. 10.2337/diabetes.51.6.1876 12031976

[64] Hotamisligil GS Shargill NS Spiegelman BM. Adipose expression of tumor necrosis factor-alpha: direct role in obesity-linked insulin resistance. Science. (1993) 259:87–91. 10.1126/science.7678183 7678183

[65] Hu FB Meigs JB Li TY Rifai N Manson JE. Inflammatory markers and risk of developing type 2 diabetes in women. Diabetes. (2004) 53:693–700. 10.2337/diabetes.53.3.693 14988254

[66] Moller DE. Potential role of TNF-alpha in the pathogenesis of insulin resistance and type 2 diabetes. Trends Endocrinol Metab. (2000) 11:212–7. 10.1016/S1043-2760(00)00272-1 10878750

[67] Lang CH Dobrescu C Bagby GJ. Tumor necrosis factor impairs insulin action on peripheral glucose disposal and hepatic glucose output. Endocrinology. (1992) 130:43–52. 10.1210/endo.130.1.1727716 1727716

[68] Donath MY Shoelson SE. Type 2 diabetes as an inflammatory disease. Nat Rev Immunol. (2011) 11:98–107. 10.1038/nri2925 21233852

[69] Liu C Feng X Li Q Wang Y Li Q Hua M. Adiponectin, TNF-alpha and inflammatory cytokines and risk of type 2 diabetes: a systematic review and meta-analysis. Cytokine. (2016) 86:100–9. 10.1016/j.cyto.2016.06.028 27498215

[70] Butcher MJ Hallinger D Garcia E Machida Y Chakrabarti S Nadler J. Association of proinflammatory cytokines and islet resident leucocytes with islet dysfunction in type 2 diabetes. Diabetologia. (2014) 57:491–501. 10.1007/s00125-013-3116-5 24429578 PMC3966210

[71] Olson NC Callas PW Hanley AJ Festa A Haffner SM Wagenknecht LE. Circulating levels of TNF-alpha are associated with impaired glucose tolerance, increased insulin resistance, and ethnicity: the Insulin Resistance Atherosclerosis Study. J Clin Endocrinol Metab. (2012) 97:1032–40. 10.1210/jc.2011-2155 22238388 PMC3319215

[72] Banerjee M Saxena M. Interleukin-1 (IL-1) family of cytokines: role in type 2 diabetes. Clin Chim Acta. (2012) 413:1163–70. 10.1016/j.cca.2012.03.021 22521751

[73] Major CD Wolf BA. Interleukin-1beta stimulation of c-Jun NH(2)-terminal kinase activity in insulin-secreting cells: evidence for cytoplasmic restriction. Diabetes. (2001) 50:2721–8. 10.2337/diabetes.50.12.2721 11723054

[74] Ammendrup A Maillard A Nielsen K Aabenhus Andersen N Serup P Dragsbaek Madsen O. The c-Jun amino-terminal kinase pathway is preferentially activated by interleukin-1 and controls apoptosis in differentiating pancreatic beta-cells. Diabetes. (2000) 49:1468–76. 10.2337/diabetes.49.9.1468 10969830

[75] Kaneto H Xu G Fujii N Kim S Bonner-Weir S Weir GC. Involvement of c-Jun N-terminal kinase in oxidative stress-mediated suppression of insulin gene expression. J Biol Chem. (2002) 277:30010–8. 10.1074/jbc.M202066200 12011047

[76] Jager J Gremeaux T Cormont M Le Marchand-Brustel Y Tanti JF. Interleukin-1beta-induced insulin resistance in adipocytes through down-regulation of insulin receptor substrate-1 expression. Endocrinology. (2007) 148:241–51. 10.1210/en.2006-0692 17038556 PMC1971114

[77] Miura K Kodama Y Inokuchi S Schnabl B Aoyama T Ohnishi H. Toll-like receptor 9 promotes steatohepatitis by induction of interleukin-1beta in mice. Gastroenterology. (2010) 139:323–334.e327. 10.1053/j.gastro.2010.03.052 20347818 PMC4631262

[78] Tilg H Moschen AR Szabo G. Interleukin-1 and inflammasomes in alcoholic liver disease/acute alcoholic hepatitis and nonalcoholic fatty liver disease/nonalcoholic steatohepatitis. Hepatology. (2016) 64:955–65. 10.1002/hep.28456 26773297

[79] Kirii H Niwa T Yamada Y Wada H Saito K Iwakura Y. Lack of interleukin-1beta decreases the severity of atherosclerosis in ApoE-deficient mice. Arterioscler Thromb Vasc Biol. (2003) 23:656–60. 10.1161/01.ATV.0000064374.15232.C3 12615675

[80] Osborn O Brownell SE Sanchez-Alavez M Salomon D Gram H Bartfai T. Treatment with an Interleukin 1 beta antibody improves glycemic control in diet-induced obesity. Cytokine. (2008) 44:141–8. 10.1016/j.cyto.2008.07.004 18723371 PMC3063393

[81] Salmenniemi U Ruotsalainen E Pihlajamaki J Vauhkonen I Kainulainen S Punnonen K. Multiple abnormalities in glucose and energy metabolism and coordinated changes in levels of adiponectin, cytokines, and adhesion molecules in subjects with metabolic syndrome. Circulation. (2004) 110:3842–8. 10.1161/01.CIR.0000150391.38660.9B 15596567

[82] Moschen AR Molnar C Enrich B Geiger S Ebenbichler CF Tilg H. Adipose and liver expression of interleukin (IL)-1 family members in morbid obesity and effects of weight loss. Mol Med. (2011) 17:840–5. 10.2119/molmed.2010.00108 21394384 PMC3146615

[83] Larsen CM Faulenbach M Vaag A Volund A Ehses JA Seifert B. Interleukin-1-receptor antagonist in type 2 diabetes mellitus. N Engl J Med. (2007) 356:1517–26. 10.1056/NEJMoa065213 17429083

[84] Kamimura D Ishihara K Hirano T. IL-6 signal transduction and its physiological roles: the signal orchestration model. Rev Physiol Biochem Pharmacol. (2003) 149:1–38. 10.1007/s10254-003-0012-2 12687404

[85] Rose-John S. IL-6 trans-signaling via the soluble IL-6 receptor: importance for the pro-inflammatory activities of IL-6. Int J Biol Sci. (2012) 8:1237–47. 10.7150/ijbs.4989 23136552 PMC3491447

[86] Schaper F Rose-John S. Interleukin-6: Biology, signaling and strategies of blockade. Cytokine Growth Factor Rev. (2015) 26:475–87. 10.1016/j.cytogfr.2015.07.004 26189695

[87] Kraakman MJ Kammoun HL Allen TL Deswaerte V Henstridge DC Estevez E Blocking IL-6 trans-signaling prevents high-fat diet-induced adipose tissue macrophage recruitment but does not improve insulin resistance. Cell Metab. (2015) 21:403–16. 10.1016/j.cmet.2015.02.006 25738456

[88] Rotter V Nagaev I Smith U. Interleukin-6 (IL-6) induces insulin resistance in 3T3-L1 adipocytes and is, like IL-8 and tumor necrosis factor-alpha, overexpressed in human fat cells from insulin-resistant subjects. J Biol Chem. (2003) 278:45777–84. 10.1074/jbc.M301977200 12952969

[89] Timper K Denson JL Steculorum SM Heilinger C Engstrom-Ruud L Wunderlich CM. IL-6 improves energy and glucose homeostasis in obesity via enhanced central IL-6 trans-signaling. Cell Rep. (2017) 19:267–80. 10.1016/j.celrep.2017.03.043 28402851

[90] Stouthard JM Oude Elferink RP Sauerwein HP. Interleukin-6 enhances glucose transport in 3T3-L1 adipocytes. Biochem Biophys Res Commun. (1996) 220:241–5. 10.1006/bbrc.1996.0389 8645290

[91] Lagathu C Bastard JP Auclair M Maachi M Capeau J Caron M. Chronic interleukin-6 (IL-6) treatment increased IL-6 secretion and induced insulin resistance in adipocyte: prevention by rosiglitazone. Biochem Biophys Res Commun. (2003) 311:372–9. 10.1016/j.bbrc.2003.10.013 14592424

[92] Pradhan AD Manson JE Rifai N Buring JE Ridker PM. C-reactive protein, interleukin 6, and risk of developing type 2 diabetes mellitus. JAMA. (2001) 286:327–34. 10.1001/jama.286.3.327 11466099

[93] Park J Ryu DR Li JJ Jung DS Kwak SJ Lee SH. MCP-1/CCR2 system is involved in high glucose-induced fibronectin and type IV collagen expression in cultured mesangial cells. Am J Physiol Renal Physiol. (2008) 295:F749–57. 10.1152/ajprenal.00547.2007 18579703

[94] Sung FL Zhu TY Au-Yeung KK Siow YL Enhanced MCP-1 expression during ischemia/reperfusion injury is mediated by oxidative stress and NF-kappa. Kidney Int B. (2002) 62:1160–70. 10.1046/j.1523-1755.2002.00577.x 12234286

[95] Chen J Guo Y Zeng W Huang L Pang Q Nie L. ER stress triggers MCP-1 expression through SET7/9-induced histone methylation in the kidneys of db/db mice. Am J Physiol Renal Physiol. (2014) 306:F916–25. 10.1152/ajprenal.00697.2012 24452638

[96] Kanda H Tateya S Tamori Y Kotani K Hiasa K Kitazawa R. MCP-1 contributes to macrophage infiltration into adipose tissue, insulin resistance, and hepatic steatosis in obesity. J Clin Invest. (2006) 116:1494–505. 10.1172/JCI26498 16691291 PMC1459069

[97] Nio Y Yamauchi T Iwabu M Okada-Iwabu M Funata M Yamaguchi M. Monocyte chemoattractant protein-1 (MCP-1) deficiency enhances alternatively activated M2 macrophages and ameliorates insulin resistance and fatty liver in lipoatrophic diabetic A-ZIP transgenic mice. Diabetologia. (2012) 55:3350–8. 10.1007/s00125-012-2710-2 22983634

[98] Ahmed SF Shabayek MI Abdel Ghany ME El-Hefnawy MH El-Mesallamy HO Role of CTRP3 CTRP9 and MCP-1 for the evaluation of T2DM associated coronary artery disease in Egyptian postmenopausal females. PLoS ONE. (2018) 13:e0208038. 10.1371/journal.pone.0208038 30557342 PMC6296499

[99] Pedersen BK Febbraio MA. Muscles, exercise and obesity: skeletal muscle as a secretory organ. Nat Rev Endocrinol. (2012) 8:457–65. 10.1038/nrendo.2012.49 22473333

[100] Bostrom P Wu J Jedrychowski MP Korde A Ye L Lo JC. A PGC1-alpha-dependent myokine that drives brown-fat-like development of white fat and thermogenesis. Nature. (2012) 481:463–8. 10.1038/nature10777 22237023 PMC3522098

[101] Roca-Rivada A Castelao C Senin LL Landrove MO Baltar J Belen Crujeiras A FNDC5/irisin is not only a myokine but also an adipokine. PLoS ONE. (2013) 8:e60563 10.1371/journal.pone.0060563 23593248 PMC3623960

[102] Huh JY Mougios V Kabasakalis A Fatouros I Siopi A Douroudos II. Exercise-induced irisin secretion is independent of age or fitness level and increased irisin may directly modulate muscle metabolism through AMPK activation. J Clin Endocrinol Metab. (2014) 99:E2154–61. 10.1210/jc.2014-1437 25119310

[103] Xiong XQ Geng Z Zhou B Zhang F Han Y Zhou YB. FNDC5 attenuates adipose tissue inflammation and insulin resistance via AMPK-mediated macrophage polarization in obesity. Metabolism. (2018) 83:31–41. 10.1016/j.metabol.2018.01.013 29374559

[104] Seale P Conroe HM Estall J Kajimura S Frontini A Ishibashi J. Prdm16 determines the thermogenic program of subcutaneous white adipose tissue in mice. J Clin Invest. (2011) 121:96–105. 10.1172/JCI44271 21123942 PMC3007155

[105] Moreno-Navarrete JM Ortega F Serrano M Guerra E Pardo G Tinahones F. Irisin is expressed and produced by human muscle and adipose tissue in association with obesity and insulin resistance. J Clin Endocrinol Metab. (2013) 98:E769–78. 10.1210/jc.2012-2749 23436919

[106] Rutti S Howald C Arous C Dermitzakis E Halban PA Bouzakri K. IL-13 improves beta-cell survival and protects against IL-1beta-induced beta-cell death. Mol Metab. (2016) 5:122–31. 10.1016/j.molmet.2015.11.003 26909320 PMC4735661

[107] Stanya KJ Jacobi D Liu S Bhargava P Dai L Gangl MR. Direct control of hepatic glucose production by interleukin-13 in mice. J Clin Invest. (2013) 123:261–71. 10.1172/JCI64941 23257358 PMC3533296

[108] Jiang LQ Franck N Egan B Sjogren RJ Katayama M Duque-Guimaraes D. Autocrine role of interleukin-13 on skeletal muscle glucose metabolism in type 2 diabetic patients involves microRNA let-7. Am J Physiol Endocrinol Metab. (2013) 305:E1359–66. 10.1152/ajpendo.00236.2013 24105413

[109] Grabstein KH Eisenman J Shanebeck K Rauch C Srinivasan S Fung V. Cloning of a T cell growth factor that interacts with the beta chain of the interleukin-2 receptor. Science. (1994) 264:965–8. 10.1126/science.8178155 8178155

[110] Sun H Liu D. Hydrodynamic delivery of interleukin 15 gene promotes resistance to high fat diet-induced obesity, fatty liver and improves glucose homeostasis. Gene Ther. (2015) 22:341–7. 10.1038/gt.2014.114 25503694

[111] Barra NG Reid S MacKenzie R Werstuck G Trigatti BL Richards C. Interleukin-15 contributes to the regulation of murine adipose tissue and human adipocytes. Obesity. (2010) 18:1601–7. 10.1038/oby.2009.445 20019685

[112] Nielsen AR Hojman P Erikstrup C Fischer CP Plomgaard P Mounier R. Association between interleukin-15 and obesity: interleukin-15 as a potential regulator of fat mass. J Clin Endocrinol Metab. (2008) 93:4486–93. 10.1210/jc.2007-2561 18697873

[113] Matthews VB Astrom MB Chan MH Bruce CR Krabbe KS Prelovsek O. Brain-derived neurotrophic factor is produced by skeletal muscle cells in response to contraction and enhances fat oxidation via activation of AMP-activated protein kinase. Diabetologia. (2009) 52:1409–18. 10.1007/s00125-009-1364-1 19387610

[114] Karczewska-Kupczewska M Kowalska I Nikolajuk A Adamska A Zielinska M Kaminska N. Circulating brain-derived neurotrophic factor concentration is downregulated by intralipid/heparin infusion or high-fat meal in young healthy male subjects. Diabetes Care. (2012) 35:358–62. 10.2337/dc11-1295 22210566 PMC3263867

[115] Pedersen BK. The diseasome of physical inactivity–and the role of myokines in muscle–fat cross talk. J Physiol. (2009) 587:5559–68. 10.1113/jphysiol.2009.179515 19752112 PMC2805368

[116] Tsuchida A Nakagawa T Itakura Y Ichihara J Ogawa W Kasuga M. The effects of brain-derived neurotrophic factor on insulin signal transduction in the liver of diabetic mice. Diabetologia. (2001) 44:555–66. 10.1007/s001250051661 11380073

[117] Wei J Shimazu J Makinistoglu MP Maurizi A Kajimura D Zong H. Glucose Uptake and Runx2 synergize to orchestrate osteoblast differentiation and bone formation. Cell. (2015) 161:1576–91. 10.1016/j.cell.2015.08.018 26091038 PMC4475280

[118] Lee NK Sowa H Hinoi E Ferron M Ahn JD Confavreux C. Endocrine regulation of energy metabolism by the skeleton. Cell. (2007) 130:456–69. 10.1016/j.cell.2007.05.047 17693256 PMC2013746

[119] Kim SP Frey JL Li Z Kushwaha P Zoch ML Tomlinson RE. Sclerostin influences body composition by regulating catabolic and anabolic metabolism in adipocytes. Proc Natl Acad Sci USA. (2017) 114:E11238–47. 10.1073/pnas.1707876115 29229807 PMC5748171

[120] Chen L Li Q Yang Z Ye Z Huang Y He M. Osteocalcin, glucose metabolism, lipid profile and chronic low-grade inflammation in middle-aged and elderly Chinese. Diabet Med. (2013) 30:309–17. 10.1111/j.1464-5491.2012.03769.x 22913521

[121] Mera P Laue K Ferron M Confavreux C Wei J Galan-Diez M. Osteocalcin signaling in myofibers is necessary and sufficient for optimum adaptation to exercise. Cell Metab. (2016) 23:1078–92. 10.1016/j.cmet.2016.05.004 27304508 PMC4910629

[122] Khrimian L Obri A Ramos-Brossier M Rousseaud A Moriceau S Nicot AS. Gpr158 mediates osteocalcin's regulation of cognition. J Exp Med. (2017) 214:2859–73. 10.1084/jem.20171320 28851741 PMC5626410

[123] Wei J Hanna T Suda N Karsenty G Ducy P. Osteocalcin promotes beta-cell proliferation during development and adulthood through Gprc6a. Diabetes. (2014) 63:1021–31. 10.2337/db13-0887 24009262 PMC3931403

[124] Otani T Mizokami A Hayashi Y Gao J Mori Y Nakamura S. Signaling pathway for adiponectin expression in adipocytes by osteocalcin. Cell Signal. (2015) 27:532–44. 10.1016/j.cellsig.2014.12.018 25562427

[125] Fulzele K Riddle RC DiGirolamo DJ Cao X Wan C Chen D. Insulin receptor signaling in osteoblasts regulates postnatal bone acquisition and body composition. Cell. (2010) 142:309–19. 10.1016/j.cell.2010.06.002 20655471 PMC2925155

[126] Shu H Pei Y Chen K Lu J. Significant inverse association between serum osteocalcin and incident type 2 diabetes in a middle-aged cohort. Diabetes Metab Res Rev. (2016) 32:867–74. 10.1002/dmrr.2808 27061949

[127] Hwang YC Jee JH Jeong IK Ahn KJ Chung HY Lee MK Circulating osteocalcin level is not associated with incident type 2 diabetes in middle-aged male subjects: mean 8.4-year retrospective follow-up study. Diabetes Care. (2012) 35:1919–24. 10.2337/dc11-2471 22773701 PMC3424992

[128] Higashibata Y Sakuma T Kawahata H Fujihara S Moriyama K Okada A. Identification of promoter regions involved in cell- and developmental stage-specific osteopontin expression in bone, kidney, placenta, and mammary gland: an analysis of transgenic mice. J Bone Miner Res. (2004) 19:78–88. 10.1359/jbmr.2004.19.1.78 14753740

[129] Kiefer FW Neschen S Pfau B Legerer B Neuhofer A Kahle M. Osteopontin deficiency protects against obesity-induced hepatic steatosis and attenuates glucose production in mice. Diabetologia. (2011) 54:2132–42. 10.1007/s00125-011-2170-0 21562757 PMC3131508

[130] Zeyda M Gollinger K Todoric J Kiefer FW Keck M Aszmann O. Osteopontin is an activator of human adipose tissue macrophages and directly affects adipocyte function. Endocrinology. (2011) 152:2219–27. 10.1210/en.2010-1328 21467192

[131] Ono M Itakura Y Nonomura T Nakagawa T Nakayama C Taiji M. Intermittent administration of brain-derived neurotrophic factor ameliorates glucose metabolism in obese diabetic mice. Metabolism. (2000) 49:129–33. 10.1016/S0026-0495(00)90988-0 10647076

[132] Sodhi CP Phadke SA Batlle D Sahai A. Hypoxia stimulates osteopontin expression and proliferation of cultured vascular smooth muscle cells: potentiation by high glucose. Diabetes. (2001) 50:1482–90. 10.2337/diabetes.50.6.1482 11375351

[133] Flo TH Smith KD Sato S Rodriguez DJ Holmes MA Strong RK. Lipocalin 2 mediates an innate immune response to bacterial infection by sequestrating iron. Nature. (2004) 432:917–21. 10.1038/nature03104 15531878

[134] Yan QW Yang Q Mody N Graham TE Hsu CH Xu Z. The adipokine lipocalin 2 is regulated by obesity and promotes insulin resistance. Diabetes. (2007) 56:2533–40. 10.2337/db07-0007 17639021

[135] Paton CM Rogowski MP Kozimor AL Stevenson JL Chang H Cooper JA Lipocalin-2 increases fat oxidation in vitro and is correlated with energy expenditure in normal weight but not obese women. Obesity. (2013) 21:E640–8. 10.1002/oby.20507 23640923

[136] Lemma S Sboarina M Porporato PE Zini N Sonveaux P Di Pompo G. Energy metabolism in osteoclast formation and activity. Int J Biochem Cell Biol. (2016) 79:168–80. 10.1016/j.biocel.2016.08.034 27590854

[137] Rashad NM El-Shal AS Etewa RL Wadea FM. Lipocalin-2 expression and serum levels as early predictors of type 2 diabetes mellitus in obese women. IUBMB Life. (2017) 69:88–97. 10.1002/iub.1594 28116808

[138] Ye Z Wang S Yang Z He M Zhang S Zhang W. Serum lipocalin-2, cathepsin S and chemerin levels and nonalcoholic fatty liver disease. Mol Biol Rep. (2014) 41:1317–23. 10.1007/s11033-013-2977-5 24390241

[139] Wang Y Lam KS Kraegen EW Sweeney G Zhang J Tso AW. Lipocalin-2 is an inflammatory marker closely associated with obesity, insulin resistance, and hyperglycemia in humans. Clin Chem. (2007) 53:34–41. 10.1373/clinchem.2006.075614 17040956

[140] Garcia-Martin A Rozas-Moreno P Reyes-Garcia R Morales-Santana S Garcia-Fontana B Garcia-Salcedo JA. Circulating levels of sclerostin are increased in patients with type 2 diabetes mellitus. J Clin Endocrinol Metab. (2012) 97:234–41. 10.1210/jc.2011-2186 22031520

[141] Urano T Shiraki M Ouchi Y Inoue S. Association of circulating sclerostin levels with fat mass and metabolic disease–related markers in Japanese postmenopausal women. J Clin Endocrinol Metab. (2012) 97:E1473–77. 10.1210/jc.2012-1218 22639287

[142] Daniele G Winnier D Mari A Bruder J Fourcaudot M Pengou Z. Sclerostin and insulin resistance in prediabetes: evidence of a cross talk between bone and glucose metabolism. Diabetes Care. (2015) 38:1509–17. 10.2337/dc14-2989 26084344

[143] Urakawa I Yamazaki Y Shimada T Iijima K Hasegawa H Okawa K. Klotho converts canonical FGF receptor into a specific receptor for FGF23. Nature. (2006) 444:770–4. 10.1038/nature05315 17086194

[144] Mirza MA Alsio J Hammarstedt A Erben RG Michaelsson K Tivesten A. Circulating fibroblast growth factor-23 is associated with fat mass and dyslipidemia in two independent cohorts of elderly individuals. Arterioscler Thromb Vasc Biol. (2011) 31:219–27. 10.1161/ATVBAHA.110.214619 20966399

[145] Silswal N Touchberry CD Daniel DR McCarthy DL Zhang S Andresen J. FGF23 directly impairs endothelium-dependent vasorelaxation by increasing superoxide levels and reducing nitric oxide bioavailability. Am J Physiol Endocrinol Metab. (2014) 307:E426–36. 10.1152/ajpendo.00264.2014 25053401 PMC4154070

[146] Hesse M Frohlich LF Zeitz U Lanske B Erben RG. Ablation of vitamin D signaling rescues bone, mineral, and glucose homeostasis in Fgf-23 deficient mice. Matrix Biol. (2007) 26:75–84. 10.1016/j.matbio.2006.10.003 17123805

[147] Streicher C Zeitz U Andrukhova O Rupprecht A Pohl E Larsson TE. Long-term Fgf23 deficiency does not influence aging, glucose homeostasis, or fat metabolism in mice with a nonfunctioning vitamin D receptor. Endocrinology. (2012) 153:1795–805. 10.1210/en.2011-1878 22294750 PMC3320267

[148] Kershaw EE Flier JS. Adipose tissue as an endocrine organ. J Clin Endocrinol Metab. (2004) 89:2548–56. 10.1210/jc.2004-0395 15181022

[149] Fasshauer M Bluher M. Adipokines in health and disease. Trends Pharmacol Sci. (2015) 36:461–70. 10.1016/j.tips.2015.04.014 26022934

[150] Perez-Perez A Vilarino-Garcia T Fernandez-Riejos P Martin-Gonzalez J Segura-Egea JJ Sanchez-Margalet V. Role of leptin as a link between metabolism and the immune system. Cytokine Growth Factor Rev. (2017) 35:71–84. 10.1016/j.cytogfr.2017.03.001 28285098

[151] Zhang Y Proenca R Maffei M Barone M Leopold L Friedman JM. Positional cloning of the mouse obese gene and its human homologue. Nature. (1994) 372:425–32. 10.1038/372425a0 7984236

[152] Friedman JM Halaas JL. Leptin and the regulation of body weight in mammals. Nature. (1998) 395:763–70. 10.1038/27376 9796811

[153] Licinio J Caglayan S Ozata M Yildiz BO de Miranda PB O'Kirwan F. Phenotypic effects of leptin replacement on morbid obesity, diabetes mellitus, hypogonadism, and behavior in leptin-deficient adults. Proc Natl Acad Sci USA. (2004) 101:4531–6. 10.1073/pnas.0308767101 15070752 PMC384781

[154] Oral EA Simha V Ruiz E Andewelt A Premkumar A Snell P. Leptin-replacement therapy for lipodystrophy. N Engl J Med. (2002) 346:570–8. 10.1056/NEJMoa012437 11856796

[155] Farooqi IS Matarese G Lord GM Keogh JM Lawrence E Agwu C. Beneficial effects of leptin on obesity, T cell hyporesponsiveness, and neuroendocrine/metabolic dysfunction of human congenital leptin deficiency. J Clin Invest. (2002) 110:1093–103. 10.1172/JCI0215693 12393845 PMC150795

[156] Wannamethee SG Lowe GD Rumley A Cherry L Whincup PH Sattar N. Adipokines and risk of type 2 diabetes in older men. Diabetes Care. (2007) 30:1200–5. 10.2337/dc06-2416 17322479

[157] Myers MG Cowley MA Munzberg H. Mechanisms of leptin action and leptin resistance. Annu Rev Physiol. (2008) 70:537–56. 10.1146/annurev.physiol.70.113006.100707 17937601

[158] Coppari R Ichinose M Lee CE Pullen AE Kenny CD McGovern RA. The hypothalamic arcuate nucleus: a key site for mediating leptin's effects on glucose homeostasis and locomotor activity. Cell Metab. (2005) 1:63–72. 10.1016/j.cmet.2004.12.004 16054045

[159] Mittendorfer B Horowitz JF DePaoli AM McCamish MA Patterson BW Klein S Recombinant human leptin treatment does not improve insulin action in obese subjects with type 2 diabetes. Diabetes. (2011) 60:1474–7. 10.2337/db10-1302 21411512 PMC3292320

[160] Polyzos SA Kountouras J Zavos C Tsiaousi E. The role of adiponectin in the pathogenesis and treatment of non-alcoholic fatty liver disease. Diabetes Obes Metab. (2010) 12:365–83. 10.1111/j.1463-1326.2009.01176.x 20415685

[161] Jung TW Lee YJ Lee MW Kim SM Jung TW. Full-length adiponectin protects hepatocytes from palmitate-induced apoptosis via inhibition of c-Jun NH2 terminal kinase. Febs J. (2009) 276:2278–84. 10.1111/j.1742-4658.2009.06955.x 19290887

[162] Polyzos SA Kountouras J Mantzoros CS. Adipokines in nonalcoholic fatty liver disease. Metabolism. (2016) 65:1062–79. 10.1016/j.metabol.2015.11.006 26725002

[163] Yamauchi T Kamon J Ito Y Tsuchida A Yokomizo T Kita S. Cloning of adiponectin receptors that mediate antidiabetic metabolic effects. Nature. (2003) 423:762–9. 10.1038/nature01705 12802337

[164] Chen YL Tao J Zhao PJ Tang W Xu JP Zhang KQ. Adiponectin receptor PAQR-2 signaling senses low temperature to promote *C. elegans* longevity by regulating autophagy. Nat Commun. (2019) 10:2602. 10.1038/s41467-019-10475-8 31197136 PMC6565724

[165] Savage DB Sewter CP Klenk ES Segal DG Vidal-Puig A Considine RV. Resistin / Fizz3 expression in relation to obesity and peroxisome proliferator-activated receptor-gamma action in humans. Diabetes. (2001) 50:2199–202. 10.2337/diabetes.50.10.2199 11574398

[166] Costandi J Melone M Zhao A Rashid S. Human resistin stimulates hepatic overproduction of atherogenic ApoB-containing lipoprotein particles by enhancing ApoB stability and impairing intracellular insulin signaling. Circ Res. (2011) 108:727–42. 10.1161/CIRCRESAHA.110.238949 21293001

[167] Banerjee RR Rangwala SM Shapiro JS Rich AS Rhoades B Qi Y. Regulation of fasted blood glucose by resistin. Science. (2004) 303:1195–8. 10.1126/science.1092341 14976316

[168] Greenhill C. Liver: asprosin - new hormone involved in hepatic glucose release. Nat Rev Endocrinol. (2016) 12:312. 10.1038/nrendo.2016.66 27125501

[169] Romere C Duerrschmid C Bournat J Constable P Jain M Xia F. Asprosin, a fasting-induced glucogenic protein hormone. Cell. (2016) 165:566–79. 10.1016/j.cell.2016.02.063 27087445 PMC4852710

[170] Duerrschmid C He Y Wang C Li C Bournat JC Romere C. Asprosin is a centrally acting orexigenic hormone. Nat Med. (2017) 23:1444–53. 10.1038/nm.4432 29106398 PMC5720914

[171] Zhang L Chen C Zhou N Fu Y Cheng X. Circulating asprosin concentrations are increased in type 2 diabetes mellitus and independently associated with fasting glucose and triglyceride. Clin Chim Acta. (2019) 489:183–8. 10.1016/j.cca.2017.10.034 29104036

[172] Zabel BA Allen SJ Kulig P Allen JA Cichy J Handel TM. Chemerin activation by serine proteases of the coagulation, fibrinolytic, and inflammatory cascades. J Biol Chem. (2005) 280:34661–6. 10.1074/jbc.M504868200 16096270

[173] Horn P Metzing UB Steidl R Romeike B Rauchfuss F Sponholz C. Chemerin in peritoneal sepsis and its associations with glucose metabolism and prognosis: a translational cross-sectional study. Crit Care. (2016) 20:39. 10.1186/s13054-016-1209-5 26873079 PMC4751629

[174] Fernandez-Trasancos A Agra RM Garcia-Acuna JM Fernandez AL Gonzalez-Juanatey JR Eiras S. Omentin treatment of epicardial fat improves its anti-inflammatory activity and paracrine benefit on smooth muscle cells. Obesity. (2017) 25:1042–9. 10.1002/oby.21832 28429889

[175] Pan HY Guo L Li Q. Changes of serum omentin-1 levels in normal subjects and in patients with impaired glucose regulation and with newly diagnosed and untreated type 2 diabetes. Diabetes Res Clin Pract. (2010) 88:29–33. 10.1016/j.diabres.2010.01.013 20129687

[176] Liang Q Zhong L Zhang J Wang Y Bornstein SR Triggle CR. FGF21 maintains glucose homeostasis by mediating the cross talk between liver and brain during prolonged fasting. Diabetes. (2014) 63:4064–75. 10.2337/db14-0541 25024372

[177] Wente W Efanov AM Brenner M Kharitonenkov A Koster A Sandusky GE. Fibroblast growth factor-21 improves pancreatic beta-cell function and survival by activation of extracellular signal-regulated kinase 1/2 and Akt signaling pathways. Diabetes. (2006) 55:2470–8. 10.2337/db05-1435 16936195

[178] Mathews ST Rakhade S Zhou X Parker GC Coscina DV Grunberger G. Fetuin-null mice are protected against obesity and insulin resistance associated with aging. Biochem Biophys Res Commun. (2006) 350:437–43. 10.1016/j.bbrc.2006.09.071 17011519

[179] Srinivas PR Wagner AS Reddy LV Deutsch DD Leon MA Goustin AS. Serum alpha 2-HS-glycoprotein is an inhibitor of the human insulin receptor at the tyrosine kinase level. Mol Endocrinol. (1993) 7:1445–55. 10.1210/mend.7.11.7906861 7906861

[180] Mori K Emoto M Yokoyama H Araki T Teramura M Koyama H. Association of serum fetuin-A with insulin resistance in type 2 diabetic and nondiabetic subjects. Diabetes Care. (2006) 29:468. 10.2337/diacare.29.02.06.dc05-1484 16443916

[181] Choi HY Hwang SY Lee CH Hong HC Yang SJ Yoo HJ. Increased selenoprotein p levels in subjects with visceral obesity and nonalcoholic Fatty liver disease. Diabetes Metab J. (2013) 37:63–71. 10.4093/dmj.2013.37.1.63 23439771 PMC3579154

[182] Misu H Takamura T Takayama H Hayashi H Matsuzawa-Nagata N Kurita S. A liver-derived secretory protein, selenoprotein P, causes insulin resistance. Cell Metab. (2010) 12:483–95. 10.1016/j.cmet.2010.09.015 21035759

[183] Misu H Ishikura K Kurita S Takeshita Y Ota T Saito Y. Inverse correlation between serum levels of selenoprotein P and adiponectin in patients with type 2 diabetes. PLoS ONE. (2012) 7:e34952. 10.1371/journal.pone.0034952 22496878 PMC3319626

[184] Yang SJ Hwang SY Choi HY Yoo HJ Seo JA Kim SG. Serum selenoprotein P levels in patients with type 2 diabetes and prediabetes: implications for insulin resistance, inflammation, and atherosclerosis. J Clin Endocrinol Metab. (2011) 96:E1325–9. 10.1210/jc.2011-0620 21677040

[185] Chawla A Nguyen KD Goh YPS. Macrophage-mediated inflammation in metabolic disease. Nat Rev Immunol. (2011) 11:738–49. 10.1038/nri3071 21984069 PMC3383854

[186] Gregor MF Hotamisligil GS. Inflammatory mechanisms in obesity. Annu Rev Immunol. (2011) 29:415–45. 10.1146/annurev-immunol-031210-101322 21219177

[187] Uysal KT Wiesbrock SM Marino MW Hotamisligil GS. Protection from obesity-induced insulin resistance in mice lacking TNF-alpha function. Nature. (1997) 389:610–4. 10.1038/39335 9335502

[188] Nov O Shapiro H Ovadia H Tarnovscki T Dvir I Shemesh E. Interleukin-1beta regulates fat-liver crosstalk in obesity by auto-paracrine modulation of adipose tissue inflammation and expandability. PLoS ONE. (2013) 8:e53626. 10.1371/journal.pone.0053626 23341960 PMC3547030

[189] Mirea AM Tack CJ Chavakis T Joosten LAB Toonen EJM. IL-1 Family Cytokine Pathways Underlying NAFLD: towards new treatment strategies. Trends Mol Med. (2018) 24:458–71. 10.1016/j.molmed.2018.03.005 29665983 PMC5939989

[190] Maedler K Sergeev P Ris F Oberholzer J Joller-Jemelka HI Spinas GA. Glucose-induced beta cell production of IL-1beta contributes to glucotoxicity in human pancreatic islets. J Clin Invest. (2017) 127:1589. 10.1172/JCI92172 12235117 PMC151125

[191] Rotter Sopasakis V Larsson BM Johansson A Holmang A Smith U. Short-term infusion of interleukin-6 does not induce insulin resistance *in vivo* or impair insulin signalling in rats. Diabetologia. (2004) 47:1879–87. 10.1007/s00125-004-1544-y 15551046

[192] Quan Y Jiang CT Xue B Zhu SG Wang X. High glucose stimulates TNFalpha and MCP-1 expression in rat microglia via ROS and NF-kappaB pathways. Acta Pharmacol Sin. (2011) 32:188–93. 10.1038/aps.2010.174 21293471 PMC4009937

[193] Mo L Shen J Liu Q Zhang Y Kuang J Pu S. Irisin is regulated by CAR in liver and is a mediator of hepatic glucose and lipid metabolism. Mol Endocrinol. (2016) 30:533–42. 10.1210/me.2015-1292 27007446 PMC5414639

[194] Rana KS Pararasa C Afzal I Nagel DA Hill EJ Bailey CJ. Plasma irisin is elevated in type 2 diabetes and is associated with increased E-selectin levels. Cardiovasc Diabetol. (2017) 16:147. 10.1186/s12933-017-0627-2 29121940 PMC5680831

[195] Almendro V Fuster G Busquets S Ametller E Figueras M Argiles JM. Effects of IL-15 on rat brown adipose tissue: uncoupling proteins and PPARs. Obesity. (2008) 16:285–9. 10.1038/oby.2007.47 18239634

[196] Barra NG Chew MV Holloway AC Ashkar AA. Interleukin-15 treatment improves glucose homeostasis and insulin sensitivity in obese mice. Diabetes Obes Metab. (2012) 14:190–3. 10.1111/j.1463-1326.2011.01495.x 21906226

[197] Duan Y Li F Wang W Guo Q Wen C Li Y. Interleukin-15 in obesity and metabolic dysfunction: current understanding and future perspectives. Obes Rev. (2017) 18:1147–58. 10.1111/obr.12567 28752527

[198] Kernie SG Liebl DJ Parada LF. BDNF regulates eating behavior and locomotor activity in mice. Embo j. (2000) 19:1290–300. 10.1093/emboj/19.6.1290 10716929 PMC305670

[199] Krabbe KS Nielsen AR Krogh-Madsen R Plomgaard P Rasmussen P Erikstrup C. Brain-derived neurotrophic factor (BDNF) and type 2 diabetes. Diabetologia. (2007) 50:431–8. 10.1007/s00125-006-0537-4 17151862

[200] Li B Lang N Cheng ZF. Serum levels of brain-derived neurotrophic factor are associated with diabetes risk, complications, and obesity: a cohort study from chinese patients with type 2 diabetes. Mol Neurobiol. (2016) 53:5492–9. 10.1007/s12035-015-9461-2 26454822

[201] Mosialou I Shikhel S Liu JM Maurizi A Luo N He Z. MC4R-dependent suppression of appetite by bone-derived lipocalin 2. Nature. (2017) 543:385–90. 10.1038/nature21697 28273060 PMC5975642

[202] Komarova SV Ataullakhanov FI Globus RK. Bioenergetics and mitochondrial transmembrane potential during differentiation of cultured osteoblasts. Am J Physiol Cell Physiol. (2000) 279:C1220–9. 10.1152/ajpcell.2000.279.4.C1220 11003602

[203] Zoch ML Abou DS Clemens TL Thorek DL Riddle RC. *In vivo* radiometric analysis of glucose uptake and distribution in mouse bone. Bone Res. (2016) 4:16004. 10.1038/boneres.2016.4 27088042 PMC4820746

[204] Esen E Chen J Karner CM Okunade AL Patterson BW Long F. WNT-LRP5 signaling induces Warburg effect through mTORC2 activation during osteoblast differentiation. Cell Metab. (2013) 17:745–55. 10.1016/j.cmet.2013.03.017 23623748 PMC3653292

[205] Ferron M Wei J Yoshizawa T Del Fattore A DePinho RA Teti A. Insulin signaling in osteoblasts integrates bone remodeling and energy metabolism. Cell. (2010) 142:296–308. 10.1016/j.cell.2010.06.003 20655470 PMC2910411

[206] Mizokami A Yasutake Y Higashi S Kawakubo-Yasukochi T Chishaki S Takahashi I. Oral administration of osteocalcin improves glucose utilization by stimulating glucagon-like peptide-1 secretion. Bone. (2014) 69:68–79. 10.1016/j.bone.2014.09.006 25230237

[207] Kiefer FW Zeyda M Gollinger K Pfau B Neuhofer A Weichhart T. Neutralization of osteopontin inhibits obesity-induced inflammation and insulin resistance. Diabetes. (2010) 59:935–46. 10.2337/db09-0404 20107108 PMC2844841

[208] Glass O Henao R Patel K Guy CD Gruss HJ Syn WK. Serum Interleukin-8, Osteopontin, and Monocyte Chemoattractant Protein 1 Are Associated With Hepatic Fibrosis in Patients With Nonalcoholic Fatty Liver Disease. Hepatol Commun. (2018) 2:1344–55. 10.1002/hep4.1237 30411081 PMC6211321

[209] Takemoto M Yokote K Nishimura M Shigematsu T Hasegawa T Kon S. Enhanced expression of osteopontin in human diabetic artery and analysis of its functional role in accelerated atherogenesis. Arterioscler Thromb Vasc Biol. (2000) 20:624–8. 10.1161/01.ATV.20.3.624 10712383

[210] Xu Y Ma X Pan X He X Xiao Y Bao Y. Correlations between serum concentration of three bone-derived factors and obesity and visceral fat accumulation in a cohort of middle aged men and women. Cardiovasc Diabetol. (2018) 17:143. 10.1186/s12933-018-0786-9 30424752 PMC6233377

[211] Ukita M Yamaguchi T Ohata N Tamura M. Sclerostin enhances adipocyte differentiation in 3T3-L1 cells. J Cell Biochem. (2016) 117:1419–28. 10.1002/jcb.25432 26553151

[212] Hu X Ma X Luo Y Xu Y Xiong Q Pan X. Associations of serum fibroblast growth factor 23 levels with obesity and visceral fat accumulation. Clin Nutr. (2018) 37:223–8. 10.1016/j.clnu.2016.12.010 28027796

[213] Gateva A Assyov Y Tsakova A Kamenov Z. Prediabetes is characterized by higher FGF23 levels and higher prevalence of Vitamin D deficiency compared to normal glucose tolerance subjects. Horm Metab Res. (2019) 51:106–11. 10.1055/a-0813-3164 30572348

[214] Wahl P Xie H Scialla J Anderson CA Bellovich K Brecklin C. Earlier onset and greater severity of disordered mineral metabolism in diabetic patients with chronic kidney disease. Diabetes Care. (2012) 35:994–1001. 10.2337/dc11-2235 22446176 PMC3329844

[215] Reyes-Garcia R Garcia-Martin A Garcia-Fontana B Morales-Santana S Rozas-Moreno P Munoz-Torres M. FGF23 in type 2 diabetic patients: relationship with bone metabolism and vascular disease. Diabetes Care. (2014) 37:e89–90. 10.2337/dc13-2235 24757249

